# Supplementary material for: Gut-directed hypnotherapy versus standard medical treatment for nausea in children with functional nausea or functional dyspepsia: protocol of a multicentre randomised trial
Source: BMJ Open. 2019 Apr 11;9(4):e024903. doi: 10.1136/bmjopen-2018-024903 (PMC6500305; doi:10.1136/bmjopen-2018-024903)
Supplement: Author's manuscript [file bmjopen-2018-024903.draft_revisions.pdf]

BMJ Open

BMJ Open is committed to open peer review. As part of this commitment we make the peer review history of every article we publish publicly available.

When an article is published we post the peer reviewers' comments and the authors' responses online. We also post the versions of the paper that were used during peer review. These are the versions that the peer review comments apply to.

The versions of the paper that follow are the versions that were submitted during the peer review process. They are not the versions of record or the final published versions. They should not be cited or distributed as the published version of this manuscript.

BMJ Open is an open access journal and the full, final, typeset and author-corrected version of record of the manuscript is available on our site with no access controls, subscription charges or pay-per-view fees (http://bmjopen.bmj.com).

If you have any questions on BMJ Open's open peer review process please email editorial.bmjopen@bmj.com

BMJ Open

Gut-Directed Hypnotherapy Versus Standard Medical Care for Nausea in Children with Functional Nausea or Functional Dyspepsia: Protocol of a Multi-Center Randomized Trial.

Journal:	*BMJ Open*
Manuscript ID	bmjopen-2018-024903
Article Type:	Protocol
Date Submitted by the Author:	27-Jun-2018
Complete List of Authors:	Browne, Pamela; Amsterdam UMC, Department of Pediatric Gastroenterology and Nutrition<br>den Hollander, Bibiche; Amsterdam UMC, Department of Pediatric Gastroenterology and Nutrition<br>Speksnijder, Esther; Amsterdam UMC, Department of Pediatric Gastroenterology and Nutrition<br>van Wering, Herbert; Amphia Ziekenhuis, Department of Pediatrics<br>Tjon a ten, Walther; Maxima Medical Center, Department of Pediatrics<br>George, Elvira; Northwest Clinics, Department of Pediatrics<br>Groeneweg, Michael; Maasstad Ziekenhuis, Department of Pediatrics<br>Bevers, Nanja; Zuyderland Medical Center, Department of Pediatrics<br>Wessels, Margreet; Rijnstate Hospital, Department of Pediatrics<br>van den Berg, Maartje; Haaglanden Medical Center, Department of Pediatrics<br>Goede, Joery; Spaarne Hospital, Department of Pediatrics<br>Teklenburg, Sarah; Isala Clinics, Department of Pediatrics<br>Frankenhuis, Carla; Amsterdam UMC, Department of Pediatric Gastroenterology and Nutrition<br>Benninga, Marc; Amsterdam UMC, Department of Pediatric Gastroenterology and Nutrition<br>Vlieger, Arine; St. Antonius Hospital, Department of Pediatrics
Keywords:	Hypnotherapy, Functional Nausea, Functional Dyspepsia, Children, Adolescents

SCHOLARONE™
Manuscripts

27 June 2018. v1.

Gut-Directed Hypnotherapy Versus Standard Medical Care for Nausea in Children with Functional Nausea or Functional Dyspepsia: Protocol of a Multi-Center Randomized Trial.

Author names and affiliations

Pamela D. Browne[1], Bibiche den Hollander[1], Esther Speksnijder[1], Herbert van Wering[2], Walther Tjon A Ten[3], Elvira K. George[4], Michael Groeneweg[5], Nanja Bevers[6], Margreet Wessels[7], Maartje van den Berg[8], Joery Goede[9], Sarah Teklenburg[10], Carla Frankenhuis[1], Marc A. Benninga[1], Arine M. Vlieger[11]

Corresponding author

Pamela D. Browne; P.D.Browne@amc.uva.nl

Present/permanent address: Department of Pediatric Gastroenterology and Nutrition, Emma Children's Hospital/Amsterdam UMC, Meibergdreef 9 (room C2-312), 1105 AZ Amsterdam, The Netherlands

1. Department of Pediatric Gastroenterology and Nutrition, Emma Children's Hospital/Amsterdam UMC, Amsterdam, Netherlands
2. Department of Pediatrics, Amphia Hospital, Breda, the Netherlands
3. Department of Pediatrics, Maxima Medical Center, Veldhoven, the Netherlands
4. Department of Pediatrics, Northwest Clinics, Alkmaar, The Netherlands
5. Department of Pediatrics, Maasstad Hospital, Rotterdam, The Netherlands
6. Department of Pediatrics, Zuyderland Medical Center, Heerlen, The Netherlands
7. Department of Pediatrics, Rijnstate Hospital, Arnhem, The Netherlands
8. Department of Pediatrics, Haaglanden Medical Center, Den Haag, The Netherlands
9. Department of Pediatrics, Spaarne Hospital, Hoofddorp, The Netherlands
10. Department of Pediatrics, Isala Clinics, Zwolle, The Netherlands
11. Department of Pediatrics, St. Antonius Hospital, Nieuwegein, The Netherlands

For peer review only

27 June 2018. v1.

Abstract

Introduction: The treatment of chronic functional nausea or nausea due to functional dyspepsia in children is generally symptomatic. Moreover, these disorders pose a risk for worse psychosocial and health outcomes in children. Hypnotherapy, by its ability to positively influence gastrointestinal and psychosocial functioning, may be an effective treatment for chronic nausea.

Methods and analysis: To test efficacy, this multi-center, parallel, randomized controlled, open label trial evaluates whether gut-directed hypnotherapy (HT) is superior to standard medical care (SMT) for reducing nausea. The study will be conducted at eleven academic and non-academic hospitals across the Netherlands. A total of hundred children (8-18 years), fulfilling the Rome IV criteria for chronic idiopathic nausea or functional dyspepsia with prominent nausea, will be randomly allocated (1:1) to receive HT or SMT. Children allocated to the HT group will receive six sessions of HT during three months, while children allocated to the SMT group will receive six sessions of SMT + supportive therapy during the same period. The primary outcome will be the difference in the proportion of children with at least 50% reduction of nausea, compared to baseline at twelve months follow-up. Secondary outcomes include the changes in abdominal pain, dyspeptic symptoms, quality of life, anxiety, depression, school absences, parental absence of work, health-care costs, and adequate relief of symptoms, measured directly after treatment, six and twelve months follow-up. If HT proves effective for reducing nausea, it may become a new treatment strategy to treat children with chronic functional nausea or functional dyspepsia with prominent nausea.

Ethics and dissemination: Results of the study will be publically disclosed to the public, without any restrictions, in peer-reviewed journal and international conferences. The study is approved by the Medical Research Ethics Committees United (MEC-U) in the Netherlands.

Registration details: Dutch trial registration number is NTR5814. Registered on 7 June 2016. http://www.trialregister.nl/trialreg/admin/rctview.asp?TC=5814

Keywords: Hypnotherapy, Functional Nausea, Functional Dyspepsia, Children, Adolescents

Words: 5972

For peer review only - http://bmjopen.bmj.com/site/about/guidelines.xhtml

27 June 2018. v1.

Article summary (strengths and limitations)

- First study to investigate the effectiveness of hypnotherapy on symptoms of nausea in children and adolescents, diagnosed with chronic idiopathic nausea or functional dyspepsia.

- A multicenter study with eleven participating academic and non-academic hospitals recruiting 100 children and adolescents.

- Long-term study follow-up of one year.

- Due to the nature of hypnotherapy, children, parents, and health care providers are not blinded for the received treatments.

1. Introduction

Chronic idiopathic nausea (CIN) and functional dyspepsia (FD) affect approximately 0.5% and 4.5-7.6% children worldwide (Robin et al., 2017), respectively, and are associated with substantial physical and psychosocial distress, school absences and decreased social functioning (Kovacic, 2013; Perez, 2007; Russell, 2015). Moreover, it has a considerable negative financial impact on health care (Brook et al., 2010). According to the Rome IV criteria, when no evidence of organic disease is found, the disorders are considered functional. Children meet the Rome IV criteria for CIN when they suffer from chronic nausea without abdominal pain, when symptoms are not related to meals, and not consistently associated with vomiting. Children are diagnosed with FD when they have chronic symptoms of epigastric pain/burning, symptoms of postprandial fullness, and/or early satiation (Hyams et al., 2016).

The treatment of CIN and FD with prominent nausea in pediatric patients is mostly symptomatic and not well defined. Most clinicians individualize the patient's medical treatment, including prokinetics, anti-emetics, antacids, and herbal products, according to their symptoms and associated comorbidities (Perez et al., 2007; Russell et al., 2015). The major disadvantage of this approach is that this treatment is symptomatic, and thus drugs often need to be used as long as patients suffer from nausea, which may take years (Madani, 2016; Rodriguez, 2013). Hence, there is a need for additional effective treatments for nausea in children with CIN or FD.

Several pathophysiologic mechanisms have been proposed to play a role in the etiology of CIN and FD, including delayed gastric emptying, impaired gastric motility and/or abnormal central nervous system processing of gastric stimuli through the gut-brain axis

For peer review only

(Perez, 2007). Additionally, there are indications that psychological factors, including anxiety and stress, may increase the severity of nausea through the gut-brain axis (Kellow et al., 2006; Oudenhove et al., 2008).

Gut-directed hypnotherapy (HT) may have the potential to reduce symptoms of nausea in children with CIN or FD. HT is a form of therapy in which a therapist, by using suggestions, can induce a hypnotic state in an individual to positively modify physiological, cognitive, and affective processes, as well as behavior in that individual (Häuser, 2016). It has been shown to be very effective in the treatment of adults and children with functional abdominal pain (Miller et al., 2015; Vlieger, 2007) and children with chemotherapy-induced nausea and vomiting (Richardson et al., 2007). Therefore we hypothesize that HT, by its ability to influence gut motility (Whorwell, 1992), psychological well-being (Gonsalkorale, 2002) and visceral hypersensitivity (Lea et al., 2003; Lowén et al., 2013; Prior, 1990), might alleviate symptoms of nausea in children with CIN or FD as well. To date, however, no studies have examined the potential effect of HT in children with CIN or FD.

The main goal of this multi-center randomized controlled trial is to evaluate the effectiveness of HT in reducing symptoms of nausea in children with CIN or FD. Six sessions of gut-direct HT will be compared with six sessions of SMT plus supportive therapy in 100 children with CIN or FD between 8 and 18 years. Additionally, we will investigate the potential influence on abdominal pain, dyspeptic symptoms, quality of life, anxiety, depression, school absences, parental absence of work and health-care costs. We expect that HT will be more effective in reducing symptoms of nausea than standard medical care. Furthermore, we expect that children receiving hypnotherapy will report more relief of symptoms (e.g. less abdominal pain, less dyspeptic symptoms), better quality of life, less symptoms of anxiety and depression, less absence from school, compared to children receiving standard medical care. We also expect that parents of children in the hypnotherapy group will report less parental absences from work and lower health care costs, compared the medical care group.

For peer review only

27 June 2018. v1.

2. Methods

2.1 Trial design

The present study in children and adolescents is a multi-center randomized controlled trial (RCT). Hundred children between ages 8 and 18 years with symptoms of nausea and fulfilling the Rome IV criteria for CIN or FD, diagnosed by their pediatrician, will be enrolled in the study. After randomization, children will receive either 6 sessions of gut-directed HT during 3 months by a qualified hypnotherapist, or 6 sessions of SMT plus supportive therapy from their pediatrician during 3 months (see Figure 1). Detailed information on the HT and SMT interventions can be found under the section '2.5. Intervention'. The additional file 1 presents the Standard Protocol Items: Recommendations for Interventional Trials (SPIRIT) checklist (see additional File 1).

2.2. Patient and Public Involvement

Patients and the public were not involved in the design of the RCT.

X

Figure 1. Standard Protocol Items: Recommendations for Interventional Trials (SPIRIT) Figure displaying the trial design and the outcome measurements. After screening for eligibility (t-1), children and parents sign the informed consent form and fill in the baseline questionnaire (t0). Children are then randomized in the hypnotherapy (HT) or standard medical care (SMT) group. Assessments take place before the start of treatment (baseline; t0), 6 weeks after the start of treatment (t1), 3 months after the start of treatment (t2), 6 months follow-up (t3) and 12 months follow-up (t4).

2.3 Recruitment

2.3.1 Recruitment procedures

Children and adolescents will be recruited in outpatient pediatric clinics of 1 academic and 10 non-academic hospitals in the Netherlands: Academic Medical Center (Amsterdam), Amphia Hospital (Breda), Maxima Medical Center (Veldhoven), Northwest Clinics (Alkmaar), Maasstad Hospital (Rotterdam), Zuyderland Medical Center (Heerlen), Rijnstate Hospital, (Arnhem), Haaglanden Medical Center (Den Haag), Spaarne Hospital (Hoofddorp), Isala Clinics (Zwolle) and St. Antonius Hospital (Nieuwegein). These centers are located in both urban and rural areas throughout the Netherlands, serving an ethnically diverse pediatric population.

For peer review only

27 June 2018. v1.

2.3.2. Participant screening

All children with symptoms of nausea and fulfilling the Rome IV criteria for CIN or FD, will undergo blood laboratory testing before inclusion, including complete blood cell count, C-reactive protein, liver function tests, creatinine, total bilirubin, and for celiac screening, amylase anti-transglutaminase antibodies and IgA testing. Additionally, urinalysis and stool analysis for parasites (*Giardia Lamblia, Entamoeba Histolytica*) and *Helicobacter pylori* antigens will be performed. The need for additional diagnostic testing, for example endoscopy to rule out eosinophilic esophagitis or 24h pH, will be left to the discretion of the treating pediatrician or pediatric gastroenterologist. The flow of the study protocol is presented in Figure 2.

X

Figure 2. The Consolidated Standards of Reporting Trials (CONSORT) flow diagram indicating the number of participants throughout the study. After eligibility screening, children are randomized in either the hypnotherapy (HT) or standard medical care (SMT) group. Follow-up measurements take place at 6 weeks after the start of treatment (t1), 3 months after the start of treatment (t2), and 6 months follow-up (t3) and 12 months follow-up (t4).

2.4. Criteria

2.4.1. Inclusion criteria

A total sample of 100 children and adolescents with CIN or FD with symptoms of nausea will be enrolled in the study. Children and adolescents can participate in this study if they meet the following inclusion criteria:

- Age 8 to 18 years at inclusion of the study
- Diagnosis of CIN or FD, with symptoms of nausea, according to Rome IV criteria (Hyams et al., 2016)
- Sufficient knowledge of the Dutch language

2.4.2. Exclusion criteria

Children will not be enrolled in the study if they meet the following exclusion criteria:

- Concomitant organic gastrointestinal disease
- Simultaneous treatment by another health care professional for symptoms of nausea
- Previously received hypnotherapy
- Intellectual disability

For peer review only

27 June 2018. v1.

2.5. Randomization, blinding, and treatment allocation

After obtaining informed consent, children are randomly allocated, by the treating pediatricians, to one of the two treatment arms: HT, given by a qualified therapist, or SMT, meaning treatment by the child's pediatrician. A computerized random-number generator will be used to randomly allocate children on a 1:1 basis with varying block sized of 2, 4 and 6. To ensure allocation concealment, central randomization will be applied and the random allocation sequence remains concealed from pediatricians enrolling children into the study. Due to the nature of HT, it is not possible to blind the participating children and health care professionals involved in the treatment of the participants.

2.6. Intervention

2.6.1. Hypnotherapy

Individual HT consist of 6 sessions of 50-60 minutes, given over a period of 3 months by a qualified hypnotherapist. Twelve hypnotherapist affiliated to the recruiting hospitals will offer the HT to children. All hypnotherapists have years of experience in performing HT in children. The hypnotherapists will use an adapted version of our previously used HT protocol (Rutten et al., 2017; Vlieger et al., 2007). The HT protocol contains exercises focusing on normalization of the gut motility, stress reduction and ego strengthening. The hypnotherapists will be instructed to use the same scripts, but are allowed to adapt the content to the child's needs. The same protocol is used for children of all ages. However, the language used will be adjusted to the child's developmental age.

In the first session, an introduction to HT will be given to the child and parents, including an explanation of what HT is and how it may help in reducing symptoms of nausea. Furthermore, the hypnotherapist will take a full history and children and parents are instructed to not talk about the nausea during the treatment period. The hypnotherapist will then start with a breathing exercise and introduce a progressive relaxation, in which children imagine floating on a big cloud. Positive suggestions to increase the child's belly comfort will also be provided. For instance, the child will be instructed to make hands warm and place both hands on the belly, imagining warmth spreading through their abdomen, and especially the stomach.

In the second session, the therapist will repeat the exercise on progressive relaxation. Additionally, the therapist will introduce an exercise focusing on reduction of anxiety and

For peer review only

stress, which is called 'the favorite place exercise'.

The third session focuses on ego strengthening and a new exercise will be introduced: 'the rainbow planet exercise' for children attending primary school, and 'the air balloon exercise' for children in secondary school. In the first exercise, children choose a personal need from a rainbow that contains different needs, for example a healthy stomach, courage, tranquility, or confidence.

In the fourth session, children are encouraged to release stress during the 'the beach without worries exercise' and additional ego strengthening suggestions are made.

The fifth session focuses on reduction of anxiety, stress and ego strengthening, as well as improved functioning of the digestive system. For the digestive system, children visualize a well working digestive system with food sliding through the stomach and bowel in a comfortable way.

In the sixth session, the previous sessions will be evaluated, remaining gastro-intestinal problems may be addressed and preceding exercises may be repeated, if requested by children. If no improvement has taken place, an exercise will be introduced in which the child is instructed to look inside the stomach to see 'what the stomach needs'.

After the first session, all children will receive a CD containing standard scripts of the exercises used during the sessions. The hypnotherapist will advise children to self-practice these exercises on a daily basis. Additionally, the therapist will encourage children to practice breathing exercises a few time a day.

2.6.2. Standard medical care + supportive therapy

In the SMT group, children will visit their treating pediatrician 6 times over a 3-month period. All pediatricians will be instructed to use the same protocol for treating symptoms of nausea. The protocol consists of a stepwise approach. In the first step, children and parents will be educated about CIN and FD, reassured that there is no structural organic underlying disease present, and dietary and lifestyle advices will be provided. Children will be advised to adhere to national practical guidelines for healthy eating by the Netherlands Nutrition Centre. Children will be recommended to avoid products containing caffeine, strong spices, citrus fruits, onions, fatty foods, and if applicable, to stop smoking. Additionally, the pediatrician will explore, together with children and parents, possible connections between stressful moments, emotional problems and complaints of nausea. If connections are

For peer review only

27 June 2018. v1.

present, the pediatrician will encourage children and parents to improve coping strategies to effectively manage stress, to reduce external stressors, and to ensure an optimal environment with sufficient relaxation. Children will also be encouraged to continue their normal daily and sport activities and to go to school, to prevent or decrease avoidance behavior.

In case this does not result in adequate relief of symptoms, the pediatrician continues with the second step. In the second step, proton pump inhibitors (PPI) in combination with domperidone will be prescribed. If this treatment is not effective in reducing symptoms the pediatrician continues with the third step, which includes the prescription of ondansetron and (dis)continuation of PPIs. If again no adequate improvement occurs, in the fourth step, Iberogast will be started for children >12 years. If children do not respond to Iberogast (>12 years) or ondansetron (<12 years), the pediatrician will continue with the fifth step and prescribe erythromycin. Finally, if previous treatment did not prove to be successful in reducing symptoms, cyproheptadine will be prescribed.

The pediatrician will evaluate each step after two to four weeks. All dosages, except for Iberogast, will be prescribed according to www.kinderformularium.nl (Dutch medical guideline for pediatricians).

In addition to the medical treatment, children will receive 6 half hour sessions of supportive therapy given by the treating pediatrician. In these sessions, the symptom progression will be discussed and patient education will be provided. Moreover, exploration of potential contributing triggers (i.e. dietary product, emotional problems and stressful events) will be evaluated together with children and parents. Supportive therapy is added to correct for the patient-therapist time in the HT group.

2.6.3. Co-interventions

After 6 sessions of HT, children visit their pediatrician to evaluate the effects of HT and, if considered necessary, to receive additional medical care.

2.7. Outcomes

2.7.1. Primary outcome

The primary outcome of the RCT is the proportion of patients with at least 50% reduction of their symptoms of nausea compare to baseline at 12 months follow-up. Children and

For peer review only - http://bmjopen.bmj.com/site/about/guidelines.xhtml

27 June 2018. v1.

adolescents report symptoms of nausea at home by using the 7-day diary. To promote retention and complete follow-up, children and parents will be reminded on regular basis, via email and phone calls, to fill in the 7-day diary and other questionnaires.

7-day diary

The 7-day diary is used by children and adolescents to score the severity, incidence and frequency of symptoms of nausea, every day during 7 consecutive days (Rutten et al., 2014; Vlieger et al., 2007, 2012).

The *severity* of nausea is assessed by the 'nausea face' analog scale, a validated tool in the pediatric population (Baxter, 2011). Children rate their degree of nausea on a day using 6-faces: face 0 indicates no nausea and face 6 indicates nausea as bad as it can be imaged. Scores on the 'nausea face' scale are transported to a daily 0-10 score. Face 0, no nausea, is scored as 0, face 1 is scored as 2, face 2 is scored as 4, face 3 is scored as 6, face 4 is scored as 8 and face 5 is scored as 10 (Baxter et al., 2011). The 'severity of nausea' (SON) score is calculated by summing up the scores of seven days, giving a maximum of score of 70 (Rutten et al., 2017; Rutten et al., 2014; Vlieger et al., 2007, 2012).

The *incidence* nausea is assessed using the 'nausea incidence' scale (NIS), adapted from the 5-point dyspepsia Likert scale (Canan, 2011; De Luca et al., 2004). It measures the incidence of symptoms during a day, where score 0 for no nausea, score 1 for 1-2 times a day, score 2 for 3-5 times a day, score 3 for intermittent complaints and score 4 for complaints were always present. The total sum of the scores of seven days indicates the severity of the nausea during a week, as experienced by the child. The maximum total score is 28 (Rutten et al., 2017; Rutten et al., 2014; Vlieger et al., 2007, 2012).

The *frequency* of symptoms of nausea is recorded in minutes/hours per day and is scored by children as 0 when there was no nausea, 1 if children had <10 minutes of nausea, 2 for 10-30 minutes of nausea, 3 for 30 minutes-2 hours of nausea, 4 for 2-4 hours of nausea and 5 if the nausea lasts >4 hours a day. The 'nausea frequency score' (NFS) is calculated by summing the scores of the seven days, with a maximum of 35 (Rutten et al., 2014; Vlieger et al., 2007, 2012).

2.7.2. Secondary outcomes

In addition to the primary outcomes, the present study investigates secondary outcomes,

For peer review only - http://bmjopen.bmj.com/site/about/guidelines.xhtml

including abdominal pain, dyspeptic symptoms, health related quality of life, anxiety, depression, school absences, parental absence of work and health-care costs. Secondary outcomes are measured at 6 weeks and 3 months after treatment, and at 6 and 12 months follow-up after the end of treatment (Figure 1).

Abdominal pain

A 7-day diary is used to assess the severity and frequency of abdominal pain, every day during 7 consecutive days. It comprises of two subscales: the abdominal pain intensity subscale (APIS) and abdominal pain frequency subscale (APFS). The APIS will be scored using an affective facial scale ranging with face 0 indicating 'no pain at all' to face 5 indicating 'the most severe pain'. No abdominal pain is scored as 0, faces 1 - 2 are scored as 1, faces 3 - 4 are scored as 2 and face 5 is scored as 3. The scores of seven days are summed up, with a maximum score of 21 (Rutten et al., 2017; Rutten et al., 2014; Vlieger et al., 2007, 2012). The APFS is recorded in minutes/hours of abdominal pain per day, with score 0 indicating no pain, score 1 if children had <10 minutes of pain, 2 for 10-30 minutes of pain, 3 for 30 minutes-2 hours of pain, 4 for 2-4 hours of pain and 5 >4 hours of pain. The scores the APFS are summed up, giving a pain frequency score of maximum 35 (Rutten et al., 2017; Rutten et al., 2014; Vlieger et al., 2007, 2012).

Dyspeptic symptoms

Severity and incidence of dyspeptic symptoms is measured using the 5-point dyspepsia Likert scale, previously used in the pediatric population (Canan, 2011; De Luca, 2004). The dyspepsia Likert scale consists of 8 gastrointestinal dyspeptic symptoms: epigastric pain, upper abdominal discomfort, retrosternal pyrosis, sour-bitter taste, halitosis, belching, nausea, and early satiety. Children score the *severity* of each symptoms during the previous two weeks on a 5-point Likert scale: score 1 'no complaints at all', score 2 'little complaints', score 3 'moderate complaints', score 4 'quite a lot of complaints' and 5 'serious complaints' (Canan et al., 2011). A higher sum score indicates more severe dyspeptic complaints (SDC score), with a maximum of 40.

Children report on the *incidence* of each of the symptoms during the previous two weeks by scoring: 1 'no complaints', 2 '1-2 times a week', 3 '3-5 times a week', 4 'intermittent complaints' and 5 'complains were always present' (Canan et al., 2011). The dyspepsia severity score (DSS) is calculated by summing up the scores, giving a maximum value of 40.

For peer review only

27 June 2018. v1.

Health related quality of life

The KIDSCREEN-52 questionnaire measures health related quality of life (QoL) in children and adolescents. The questionnaire has been shown a valid tool in the Dutch pediatric population (Ravens-Sieberer et al., 2008; The Kidscreen Group Europe, 2006). The KIDSCREEN-52 consists of items on ten dimensions related to QoL on a 5-point Likert scale: moods and emotions, self-perception, relations with parents and home life, autonomy, physical well-being, psychological well-being, school environment, social support and peers, social acceptance (bullying) and financial resources. For each individual dimension, Rasch scores are computed from the individual items. These are then transformed into T-values: higher T-values indicate a better health related QoL and well-being.

Anxiety and depression

Anxiety and depression are evaluated using the Revised Anxiety and Depression Scale-short version (RCADS-25). This questionnaire has been previously validated in the Dutch pediatric population (Muris, 2002). The RCADS-25 consists of five subscales measuring symptoms of generalized anxiety disorders, separation anxiety disorder, social phobia, panic disorder and major depressive disorder. Each subscale contains five items and scales range from 0 (never) to 3 (always). The total score on anxiety or depression is the sum of the items measuring symptoms of anxiety and depressive symptoms, respectively. Higher scores indicate more symptoms of anxiety or depression.

Cost effectiveness/cost utility

The Health Utility Index Mark 3 (HUI) will be used in the cost-utility and cost-effectiveness analysis. The HUI-3 is a multi-attribute utility measure of health status in children as reported by parents. Proxy measurements of parents for health status of children are justifiable, as some children may be too young to provide reliable and valid information about their own health status (Eiser & Morse, 2001; Feeny et al., 2002; Tarride et al., 2010). The questionnaire consists of eight dimensions of health status: vision, hearing, speech, ambulation, dexterity, emotion, cognition, and pain with 5 or 6 levels per attribute, which scales varying from highly impaired to normal. Health utilities of 1 indicate perfect health, whereas 0 indicates death. The Quality adjusted life years (QALY) will be calculated by multiplying the sum of the utility of health states by the time in between measurements.

Work absenteeism by parents and school absenteeism by children

An adapted version of the Dutch Health and Labor Questionnaire (HLQ) will be used to

For peer review only - http://bmjopen.bmj.com/site/about/guidelines.xhtml

27 June 2018. v1.

measure work absenteeism by parents, school absenteeism by children, and indirect costs of health care utilization (Van Roijen, 1996). This adapted version contains three items. Parents indicate whether their child has been absent from school due to complaints of nausea, and if yes, the amount of hours per week. For work absenteeism by parents, parents indicate the number of hours they worked less on average because of their child's symptoms of nausea. For the indirect costs of health care utilization, parents indicate additional costs they had due to symptoms of nausea of their child over the past four weeks.

Somatization

The Children's Somatization Inventory (CSI) measures the extent to which children and adolescents experience somatic symptoms. The questionnaire has been shown a valid and reliable self-report instrument in the pediatric population (Meesters, 2003). The CSI consists of 35 items and on a 5-point Likert scale (0 = not at all to 4 = a whole lot) and children rate the extent to which they experienced somatic symptoms in the previous two weeks. The total score is calculated by summing up the 35 items, with higher scores indicating higher intensity of somatic complaints experienced by the child.

In order to calculate a separate CSI-score for non-gastrointestinal (GI) symptoms, 7 items on GI-symptoms (nausea, constipation, diarrhea, epigastric and abdominal pain, vomiting and bloating) are left out. The total score of somatic symptoms without GI-symptoms is calculated by summing up the scores of non-GI symptoms, with higher scores reflecting higher intensity of non-GI somatic complaints.

Adequate relief

Parents and children will be asked whether adequate relief of symptoms of nausea has occurred, using a dichotomous scale (yes/no). Adequate relief has been previously used as an endpoint in clinical trials assessing hypnotherapy in children and adolescents (Rutten et al., 2017) and has been shown a valid outcome measure for functional gastrointestinal disorders (Mangel et al., 1998).

2.8. Sample size calculation

The primary outcome of the RCT is the proportion of patients with at least 50% reduction of their symptoms of nausea compare to baseline at 12 months follow-up. Based on our pilot study (Vlieger, A. M. "A pilot-study of hypnotherapy as a treatment for functional nausea in children") and the success percentages in studies using hypnotherapy in adults with

For peer review only - http://bmjopen.bmj.com/site/about/guidelines.xhtml

27 June 2018. v1.

functional dyspepsia (Calvert, 2002) and in pediatric cancer patients (Richardson et al., 2007), we expect that 80% of the children in the hypnotherapy group will have >50% reduction of their symptoms of nausea after one year. In the standard medical care group, we anticipate that 50% of the children will have >50% reduction of their symptoms of nausea after one year. Based on these expected proportions, 45 children per group will be needed to achieve a power of 80% with a one-sided significance level of 5%. Accounting for a 10% dropout, 100 children will be included in this study. If a child is prematurely withdrawn from the study, he/she will not be replaced; data will be analyzed according to the intention to treat analysis.

2.9. Statistical analysis

2.9.1. Primary outcome

Outcomes will be analyzed according to the intention to treat (ITT) analysis. For the primary outcome, the Chi-square test will be used to compare the proportions of patients with >50% reduction of symptoms of nausea (i.e. severity, incidence and frequency of nausea) after 12 months follow-up between the two groups (HT versus SMT). For all analysis, the significance level for statistical analysis is set at $\alpha = 0.05$. Multiple imputation will be applied to deal with cases of missing data.

2.9.2. Secondary outcomes

For the secondary outcomes, including the potential influence on abdominal pain, dyspeptic symptoms, health related quality of life, anxiety, depression, work absenteeism by parents, school absenteeism by children, somatization and adequate relief, the Student's *t*-test will be used for means of normally distributed data, the Mann–Whitney U test for nonparametric data and the Chi-square test to compare proportions. To calculate the cost-effectiveness, cost-utility and cost-effectiveness ratios will be calculated for the extra costs per child with >50% reduction of symptoms of nausea and the extra costs per QALY.

As secondary analysis, the proportion of patients with >50% reduction of their nausea (severity, incidence and frequency) after treatment, 6 months and 12 months follow up will be compared between groups using multivariate logistic regression correcting for age and center.

For peer review only - http://bmjopen.bmj.com/site/about/guidelines.xhtml

27 June 2018. v1.

3. Amendments

Prior to implementation, amendments will be examined and approved by the METC. The sponsor will only record non-substantial amendments.

4. Data monitoring

Hypnotherapy is usually well tolerated in children, without significant side effects. In our previous trials only a minority of children reported some dizziness, mostly during or directly after the end of the first session (Rutten et al., 2014; Vlieger et al., 2007). In case children experience dizziness, they will be advised to execute the remaining sessions in a sitting position instead of a supine position. Children assigned to the SMT group will receive standard medical treatment, including drugs that have been either registered for children, or of which side effects are limited and well known. For these reasons, no Data Monitoring Safety Board will be established.

Study auditing will be accomplished by periodic visits to the participating centers, and by email and telephone contact with local investigators, to ensure the study protocol is being complied with and to discuss any problems that might have arisen.

5. Potential harms

In accordance to the legal requirements in the Netherlands (article 10, subsection 1, WMO), the investigator will inform the subjects and the reviewing accredited METC if harmful events occur. When there are indications that the disadvantage of participation may be significantly greater than was described in the research proposal, the study will be suspended pending further review by the accredited METC. However, the study will not be suspended if it would jeopardize participating children's health.

6. Ancillary and post-trial care

In accordance with Article 7 WMO and the Measure regarding Compulsory Insurance for Clinical Research in Humans of 23th June 200th, the sponsor has liability insurance, which provides cover for potential damage to children caused by the study.

7. Data storage

The related information on paper, including 7-day diaries, will be securely stored in a locked file cabinet with limited access. Online questionnaire data will be securely stored using the University's password-protected access systems. Only the main researchers will be given full

For peer review only

27 June 2018. v1.

access to the questionnaire data. All records that contain names will be saved in one file, which will be password protected and only accessible to the main researchers.

8. Dissemination policy

The researchers will communicate trial results to the public, health care providers, and other relevant groups via reports, and by publishing in peer-reviewed journals. Negative as well as positive results will be published. The results will be shared with participating children and parents after completion of the trial. All authors who provided substantial contributions to the conduct, interpretation, and reporting of the results will be granted authorship on the final trial report.

9. Discussion and conclusion

Chronic nausea is a highly disabling symptom for children with CIN or FD, and poses a risk for negative health outcomes and decreased psychosocial functioning (Kovacic et al., 2013; Perez & Youssef, 2007; Russell et al., 2015). To date large randomized placebo controlled trials evaluating the effect of any drug in children with either CIN or FD are lacking. Current medical treatment aiming to relieve nausea is experienced based, however these treatments are symptomatic and often used for months or years. For these reasons, new effective treatment options to reduce nausea in children with CIN or FD are warranted.

There are indications that HT can decrease symptoms of functional nausea and dyspepsia in adults (Calvert et al., 2002), and functional abdominal pain (FAP) (Vlieger et al., 2007) and chemotherapy induced nausea in children (Richardson, 2007). Calvert et al. (2002) found that adult patients with FD receiving 12 sessions of HT had significantly less dyspeptic symptoms (59%, N=26) compared to patients receiving medical treatment (33%, N=29) (p=0.02). These beneficial effects were maintained for more than a year: 56 weeks after the first treatment, 73% of the patients in the HT group reported symptom improvement compared to 43% in the medical treatment group (p<0.01). In children with FAP, Vlieger (2007) found that HT was highly superior compared to standard medical care to reduce abdominal pain. At one year follow-up, 85% of the children in the HT group (N=26) were in clinical remission compared to 85% of the children in the SMT group (N=24) (p<0.001). Additionally, a systematic review including six RCTs evaluating the effectiveness of

For peer review only - http://bmjopen.bmj.com/site/about/guidelines.xhtml

27 June 2018. v1.

hypnotherapy to reduce chemotherapy-induced nausea found hypnotherapy was most effective when compared with standard medical care to reduce complaints (*D*=0.99) (Richardson, 2007).

The present study is the first study to investigate the effectiveness of HT on symptoms of nausea in children and adolescents diagnosed with CIN or FD, according to the Rome IV criteria. If shown effective, it may provide an additional treatment option for children with CIN or FD.

The study has several strengths. The first strength is that pediatricians from eleven different hospitals throughout the Netherlands will recruit all children and adolescents. The hospitals, both an academic center and teaching hospitals, serve an ethnic and socio-economic diverse population of children and adolescents. This recruitment method has two advantages: first, it may reduce response bias to the intervention. It has previously been reported that patients from primary and secondary level care may have different responses to treatment (Veldhuyzen van Zanten et al., 1999). All children included in the present study receive secondary level care. The second advantage is that the multicenter design of the study will increase generalizability of the trial outcomes.

Another strength of the study is the long-term follow up of one year, which allows us to properly compare the potential effectiveness of HT with SMT. It has been known that the severity of functional gastrointestinal symptoms in children varies over time (Miele et al., 2004) and a continuous improvement in symptoms is often reported in children receiving hypnotherapy (Vlieger et al., 2007).

This study also has several limitations. The first limitation is that children, parents, investigators and health care providers are not blinded for the received treatments, which is not possible due the nature of HT. Several solutions will be applied to limit the risk of bias. First, to minimize performance bias, pediatricians and hypnotherapists will follow treatment guidelines to prevent any use of additional or alternative forms of care during the study period that may influence treatment outcomes. Second, to reduce the risk of detection bias, children and adolescents use reliable outcome measures and record symptoms themselves at home. Moreover, children and adolescents record symptoms of nausea for seven consecutive days, which corrects for individual variability of symptoms over time. Third, the endpoints of the study will be preregistered and the data analyst will be blinded during the

For peer review only

27 June 2018. v1.

analyses of data.

The second limitation is that the SMT provided by pediatricians in this study may not reflect usual clinical practice. In the present study, children and adolescents receive longer and more intensive medical therapy from their pediatrician compared to the real medical practice situation. However, previous studies indicate that patient-provider interactions can largely influence gastrointestinal treatment outcomes (Dossett et al., 2015). Therefore, it is important to control for the time spent per patient in the SMT group.

If the results of this study show that HT given by a therapist is comparable or slightly more effective than medical treatment provided by pediatricians, HT may become a new treatment strategy to help children with CIN or FD. Furthermore, as HT is presumably less costly than treatments by a specialist it may also decrease health care costs.

Abbreviations

APIS: Abdominal Pain Intensity Subscale; **APFS:** abdominal pain frequency subscale**; CIN:** Chronic Idiopathic Nausea**; CSI:** Children's Somatization Inventory; **FAP**: Functional Abdominal Pain**; FD:** Functional Dyspepsia**; FGID:** Functional Gastrointestinal Disorders; **GI:** gastrointestinal; **HLQ**: Health and Labor Questionnaire; **HT:** Hypnotherapy; **HUI:** Health Utility Index Mark 3**; ITT:** Intention to Treat Analysis; **MEC-U:** Medical Research Ethics Committees United; **NIS**: Nausea Incidence Scale; **NFS:** Nausea Frequency Score; **PPI:** Proton Pump Inhibitors; **QALY:** Quality Adjusted Life Years**; QoL:** Health related Quality of Life**; RCADS-25:** Revised Anxiety and Depression Scale-short version**; RCT:** Randomized Controlled Trial; **REC**: Research Ethical Committees**; SMT:** Standard Medical Care plus supportive therapy; **SON**: Severity Of Nausea; **WMO**: Medical Research Involving Human Subjects Act

Acknowledgements

None

Competing interests

The authors declare no competing interests.

Funding

The Christine Bader Foundation and Gastrointestinal and Liver Foundation supported this work. These funding sources had no role in the design of this study. They will not have any role during the collection of data, analyses or submission of the results.

For peer review only

27 June 2018. v1.

Author contributions

AV is the principle investigator, designed the study, wrote the protocol, supervised the trial and supervised writing the manuscript. PB provided adjustments to the protocol, drafted the manuscript and coordinated the trial. MB critically revised the protocol, supervised the trial and supervised writing the manuscript. All other authors participated in patient recruitment and/or treatment, read and approved the manuscript.

Trial status

Recruitment started in September 2016 and is ongoing. In October 2016, the first participant enrolled. Currently, 92 children are participating in the study (18 of them have completed the study protocol).

Ethics approval and consent to participate

This RCT was approved by the Medical Research Ethics Committees United (MEC-U) in Nieuwegein, the Netherlands (file number: NL51167.100.15). The study will be conducted in accordance with the Medical Research Involving Human Subjects Act (WMO) and conferring to the principles of the Declaration of Helsinki (64th WMA General Assembly, Fortaleza, Brazil, October 2013). The study will follow the conduct code concerning resistance in minors who participate in clinical trials as defined by the Dutch Pediatric Society. Informed consent will be asked from parents/guardians of children <12 years of age. In children and adolescents ≥12 years of age informed consent will be asked from the parents-guardians and the children and adolescents. In the event of amendments of the protocol, relevant research ethical committees (RECs) will be informed. Results of the study will be publically disclosed in a peer-reviewed journal, without any restrictions; both positive as well as negative results will be published.

5 peer reviewers suggested

Miguel Saps (msaps@childrensmemrial.org), Miranda van Tilburg (Tilburg@med.unc.edu), Peter Whorwell (peter.whorwell@smuht.nwest.nhs.uk), Olafur Palsson (opalsson@med.unc.edu), Juliette Rutten (j.m.rutten@amc.uva.nl), Emma Louise Calvert.

For peer review only

27 June 2018. v1.

References

Baxter, A. L., Watcha, M. F., Baxter, W. V., Leong, T., & Wyatt, M. M. (2011). Development and validation of a pictorial nausea rating scale for children. *Pediatrics*, *127*(6), e1542–9. http://doi.org/10.1542/peds.2010-1410

Brook, R. A., Kleinman, N. L., Choung, R. S., Melkonian, A. K., Smeeding, J. E., & Talley, N. J. (2010). Functional Dyspepsia Impacts Absenteeism and Direct and Indirect Costs. *Clinical Gastroenterology and Hepatology*, *8*(6), 498–503. http://doi.org/10.1016/j.cgh.2010.03.003

Calvert, E. L., Houghton, L. A., Cooper, P., Morris, J., & Whorwell, P. J. (2002). Long-term improvement in functional dyspepsia using hypnotherapy. *Gastroenterology*, *123*(6), 1778–1785. http://doi.org/10.1053/gast.2002.37071

Canan, O., Ozcay, F., Ozbay-Hosnut, F., Yazici, C., & Bilezikci, B. (2011). Value of the Likert dyspepsia scale in differentiation of functional and organic dyspepsia in children. *Journal of Pediatric Gastroenterology and Nutrition*, *52*(4), 392–8. http://doi.org/10.1097/MPG.0b013e3181fea0a9

De Luca, L., Zagari, R. M., Pozzato, P., Fiorini, T., Ricciardiello, L., Martuzzi, C., … Veldhuyzen van Zanten, S. J. O. (2004). Measuring dyspepsia: a new severity index validated in Bologna. *Digestive and Liver Disease*, *36*(12), 806–810. http://doi.org/10.1016/j.dld.2004.07.010

Dossett, M. L., Mu, L., Davis, R. B., Bell, I. R., Lembo, A. J., Kaptchuk, T. J., & Yeh, G. Y. (2015). Patient-Provider Interactions Affect Symptoms in Gastroesophageal Reflux Disease: A Pilot Randomized, Double-Blind, Placebo-Controlled Trial. *PLOS ONE*, *10*(9), e0136855. http://doi.org/10.1371/journal.pone.0136855

Eiser, C., & Morse, R. (2001). The measurement of quality of life in children: past and future perspectives. *Journal of Developmental and Behavioral Pediatrics : JDBP*, *22*(4), 248–56. Retrieved from http://www.ncbi.nlm.nih.gov/pubmed/11530898

Feeny, D., Furlong, W., Torrance, G. W., Goldsmith, C. H., Zhu, Z., DePauw, S., … Boyle, M. (2002). Multiattribute and single-attribute utility functions for the health utilities index mark 3 system. *Medical Care*, *40*(2), 113–28. Retrieved from http://www.ncbi.nlm.nih.gov/pubmed/11802084

Gonsalkorale, W. M., Houghton, L. A., & Whorwell, P. J. (2002). Hypnotherapy in irritable bowel syndrome: a large-scale audit of a clinical service with examination of factors influencing responsiveness. *The American Journal of Gastroenterology*, *97*(4), 954–961. http://doi.org/10.1111/j.1572-0241.2002.05615.x

Häuser, W., Hagl, M., Schmierer, A., & Hansen, E. (2016). The Efficacy, Safety and Applications of Medical Hypnosis. *Deutsches Arzteblatt International*, *113*(17), 289–96. http://doi.org/10.3238/arztebl.2016.0289

Hyams, J. S., Di Lorenzo, C., Saps, M., Shulman, R. J., Staiano, A., & van Tilburg, M. (2016). Childhood Functional Gastrointestinal Disorders: Child/Adolescent. *Gastroenterology*, *150*(6), 1456–1468.e2. http://doi.org/10.1053/j.gastro.2016.02.015

Kellow, J. E., Azpiroz, F., Delvaux, M., Gebhart, G. F., Mertz, H. R., Quigley, E. M. M., & Smout, A. J. P. M. (2006). Applied Principles of Neurogastroenterology: Physiology/Motility Sensation. *Gastroenterology*, *130*(5), 1412–1420. http://doi.org/10.1053/j.gastro.2005.08.061

Kovacic, K., Williams, S., Li, B. U. K., Chelimsky, G., & Miranda, A. (2013). High prevalence of nausea in children with pain-associated functional gastrointestinal disorders: are Rome criteria applicable? *Journal of Pediatric Gastroenterology and Nutrition*, *57*(3), 311–5.

For peer review only

27 June 2018. v1.

http://doi.org/10.1097/MPG.0b013e3182964203

Lea, R., Houghton, L. A., Calvert, E. L., Larder, S., Gonsalkorale, W. M., Whelan, V., … Whorwell, P. J. (2003). Gut-focused hypnotherapy normalizes disordered rectal sensitivity in patients with irritable bowel syndrome. *Alimentary Pharmacology & Therapeutics*, *17*(5), 635–42. Retrieved from http://www.ncbi.nlm.nih.gov/pubmed/12641511

Lowén, M. B. O., Mayer, E. A., Sjöberg, M., Tillisch, K., Naliboff, B., Labus, J., … Walter, S. A. (2013). Effect of hypnotherapy and educational intervention on brain response to visceral stimulus in the irritable bowel syndrome. *Alimentary Pharmacology & Therapeutics*, *37*(12), 1184–1197. http://doi.org/10.1111/apt.12319

Madani, S., Cortes, O., & Thomas, R. (2016). Cyproheptadine Use in Children With Functional Gastrointestinal Disorders. *Journal of Pediatric Gastroenterology and Nutrition*, *62*(3), 409–413. http://doi.org/10.1097/MPG.0000000000000964

Mangel, A., Hahn, B., Heath, A., Northcutt, A., Kong, S., Dukes, G., & McSorley, D. (1998). Adequate Relief as an Endpoint in Clinical Trials in Irritable Bowel Syndrome. *Journal of International Medical Research*, *26*(2), 76–81. http://doi.org/10.1177/030006059802600203

Meesters, C., Muris, P., Ghys, A., Reumerman, T., & Rooijmans, M. (2003). The Children's Somatization Inventory: further evidence for its reliability and validity in a pediatric and a community sample of Dutch children and adolescents. *Journal of Pediatric Psychology*, *28*(6), 413–22. Retrieved from http://www.ncbi.nlm.nih.gov/pubmed/12904453

Miele, E., Simeone, D., Marino, A., Greco, L., Auricchio, R., Novek, S. J., & Staiano, A. (2004). Functional Gastrointestinal Disorders in Children: An Italian Prospective Survey. Retrieved from http://pediatrics.aappublications.org/content/pediatrics/114/1/73.full.pdf?casa_token=RmcLJvdUTNYAAAAA:lgptkif02xEXwN1OJtqSVQxq7shfjbdFZloAjUVlbRX8oaJKTbIpimCFbzSHpm91pl__xoFRrnk

Miller, V., Carruthers, H. R., Morris, J., Hasan, S. S., Archbold, S., & Whorwell, P. J. (2015). Hypnotherapy for irritable bowel syndrome: an audit of one thousand adult patients. *Alimentary Pharmacology & Therapeutics*, *41*(9), 844–855. http://doi.org/10.1111/apt.13145

Muris, P., Meesters, C., & Schouten, E. (2002). A brief questionnaire of DSM-IV-defined anxiety and depression symptoms among children. *Clinical Psychology & Psychotherapy*, *9*(6), 430–442. http://doi.org/10.1002/cpp.347

Oudenhove, L. Van, Vandenberghe, J., Geeraerts, B., Vos, R., Persoons, P., Fischler, B., … Tack, J. (2008). Determinants of symptoms in functional dyspepsia: gastric sensorimotor function, psychosocial factors or somatisation? http://doi.org/10.1136/gut.2008.158162

Perez, M. E., & Youssef, N. N. (2007). Dyspepsia in childhood and adolescence: insights and treatment considerations. *Current Gastroenterology Reports*, *9*(6), 447–55. Retrieved from http://www.ncbi.nlm.nih.gov/pubmed/18377794

Perez, P. F., Doré, J., Leclerc, M., Levenez, F., Benyacoub, J., Serrant, P., … Donnet-Hughes, A. (2007). Bacterial imprinting of the neonatal immune system: lessons from maternal cells? *Pediatrics*, *119*(3), e724–32. http://doi.org/10.1542/peds.2006-1649

Prior, A., Colgan, S. M., & Whorwell, P. J. (1990). Changes in rectal sensitivity after hypnotherapy in patients with irritable bowel syndrome. *Gut*, *31*(8), 896–8. Retrieved from http://www.ncbi.nlm.nih.gov/pubmed/2387513

For peer review only - http://bmjopen.bmj.com/site/about/guidelines.xhtml

27 June 2018. v1.

Ravens-Sieberer, U., Gosch, A., Rajmil, L., Erhart, M., Bruil, J., Power, M., … KIDSCREEN Group. (2008). The KIDSCREEN-52 Quality of Life Measure for Children and Adolescents: Psychometric Results from a Cross-Cultural Survey in 13 European Countries. *Value in Health*, *11*(4), 645–658. http://doi.org/10.1111/j.1524-4733.2007.00291.x

Richardson, J., Smith, J. E., McCall, G., Richardson, A., Pilkington, K., & Kirsch, I. (2007). Hypnosis for nausea and vomiting in cancer chemotherapy: a systematic review of the research evidence. *Eur J Cancer Care (Engl)*, *16*(5), 402–412. http://doi.org/10.1111/j.1365-2354.2006.00736.x

Robin, S. G., Keller, C., Zwiener, R., Hyman, P. E., Nurko, S., Saps, M., … L van Tilburg, M. A. (2017). Prevalence of Pediatric Functional Gastrointestinal Disorders Utilizing the Rome IV Criteria. http://doi.org/10.1016/j.jpeds.2017.12.012

Rodriguez, L., Diaz, J., & Nurko, S. (2013). Safety and Efficacy of Cyproheptadine for Treating Dyspeptic Symptoms in Children. *The Journal of Pediatrics*, *163*(1), 261–267. http://doi.org/10.1016/J.JPEDS.2012.12.096

Russell, A., Sherman, A. L., & Walker, L. S. (2015). 620 Nausea Complicating Recurrent Abdominal Pain in Childhood Predicts Functional GI Disorders, Disability, Depression and Anxiety in Young Adulthood: Results of a Prospective Cohort Study. *Gastroenterology*, *148*(4), S–122. http://doi.org/10.1016/S0016-5085(15)30423-6

Rutten, J. M. T. M., Vlieger, A. M., Frankenhuis, C., George, E. K., Groeneweg, M., Norbruis, O. F., … Benninga, M. A. (2017). Home-Based Hypnotherapy Self-exercises vs Individual Hypnotherapy With a Therapist for Treatment of Pediatric Irritable Bowel Syndrome, Functional Abdominal Pain, or Functional Abdominal Pain Syndrome. *JAMA Pediatrics*, *171*(5), 470. http://doi.org/10.1001/jamapediatrics.2017.0091

Rutten, J. M., Vlieger, A. M., Frankenhuis, C., George, E. K., Groeneweg, M., Norbruis, O. F., … Benninga, M. A. (2014). Gut-directed hypnotherapy in children with irritable bowel syndrome or functional abdominal pain (syndrome): a randomized controlled trial on self exercises at home using CD versus individual therapy by qualified therapists. *BMC Pediatrics*, *14*(1), 140. http://doi.org/10.1186/1471-2431-14-140

Tarride, J.-E., Burke, N., Bischof, M., Hopkins, R. B., Goeree, L., Campbell, K., … Goeree, R. (2010). A review of health utilities across conditions common in paediatric and adult populations. *Health and Quality of Life Outcomes*, *8*, 12. http://doi.org/10.1186/1477-7525-8-12

The Kidscreen Group Europe. (2006). *The KIDSCREEN questionnaires: Quality of life questionnaires for children and adolescents. Lengerich*.

Van Roijen, L., Essink-bot, M.-L., Koopmanschap, M. A., Bonsel, G., & Rutten, F. F. H. (1996). Labor and Health Status in Economic Evaluation of Health Care: The Health and Labor Questionnaire. *International Journal of Technology Assessment in Health Care*, *12*(03), 405–415. http://doi.org/10.1017/S0266462300009764

Veldhuyzen van Zanten, S. J., Talley, N. J., Bytzer, P., Klein, K. B., Whorwell, P. J., & Zinsmeister, A. R. (1999). Design of treatment trials for functional gastrointestinal disorders. *Gut*, *45 Suppl 2*(suppl 2), II69–77. http://doi.org/10.1136/GUT.45.2008.II69

Vlieger, A. M., Menko-Frankenhuis, C., Wolfkamp, S. C. S., Tromp, E., & Benninga, M. A. (2007). Hypnotherapy for children with functional abdominal pain or irritable bowel syndrome: a randomized controlled trial. *Gastroenterology*, *133*(5), 1430–6. http://doi.org/10.1053/j.gastro.2007.08.072

For peer review only

27 June 2018. v1.

Vlieger, A. M., Rutten, J. M. T. M., Govers, A. M. A. P., Frankenhuis, C., & Benninga, M. A. (2012). Long-term follow-up of gut-directed hypnotherapy vs. standard care in children with functional abdominal pain or irritable bowel syndrome. *The American Journal of Gastroenterology*, *107*(4), 627–31. http://doi.org/10.1038/ajg.2011.487

Whorwell, P. J., Houghton, L. A., Taylor, E. E., & Maxton, D. G. (1992). Physiological effects of emotion: assessment via hypnosis. *Lancet (London, England)*, *340*(8811), 69–72. Retrieved from http://www.ncbi.nlm.nih.gov/pubmed/1352012

World Medical Association Declaration of Helsinki Ethical Principles for Medical Research Involving Human Subjects. (1964). *WMA General Assembly, Somerset West, Republic of South Africa*. Retrieved from https://www.wma.net/wp-content/uploads/2016/11/DoH-Oct2013-JAMA.pdf

For peer review only

	Enrolment	Allocation	During treatment		Follow-up	
**TIMEPOINT**	Before treatment (t-1)	Start of treatment (t0)	6 weeks after the start of treatment (t1)	3 months after the start of treatment (t2)	6 months follow-up (t3)	12 months follow-up (t4)
**ENROLMENT:**						
**Eligibility screen**	X					
**Informed consent**	X					
**Randomization**	X					
**INTERVENTIONS:**						
**Hypnotherapy group (HT)**						
**Standard medical care group (SMT)**						
**ASSESSMENTS:**						
Nausea and abdominal pain	X		X	X	X	X
Dyspeptic symptoms		X		X	X	X
Health related quality of life		X		X	X	X
Anxiety and Depression		X		X	X	X
Cost effectiveness/cost utility		X		X	X	X
Work absenteeism by parents and school absenteeism by children		X		X	X	X
Somatization		X		X	X	X
Adequate relief					X	X

Figure 1. Standard Protocol Items: Recommendations for Interventional Trials (SPIRIT) Figure displaying the trial design and the outcome measurements. After screening for eligibility (t-1), children and parents sign the informed consent form and fill in the baseline questionnaire (t0). Children are then randomized in the hypnotherapy (HT) or standard medical care (SMT) group. Assessments take place before the start of treatment (baseline; t0), 6 weeks after the start of treatment (t1), 3 months after the start of treatment (t2), 6 months follow-up (t3) and 12 months follow-up (t4).

215x279mm (300 x 300 DPI)

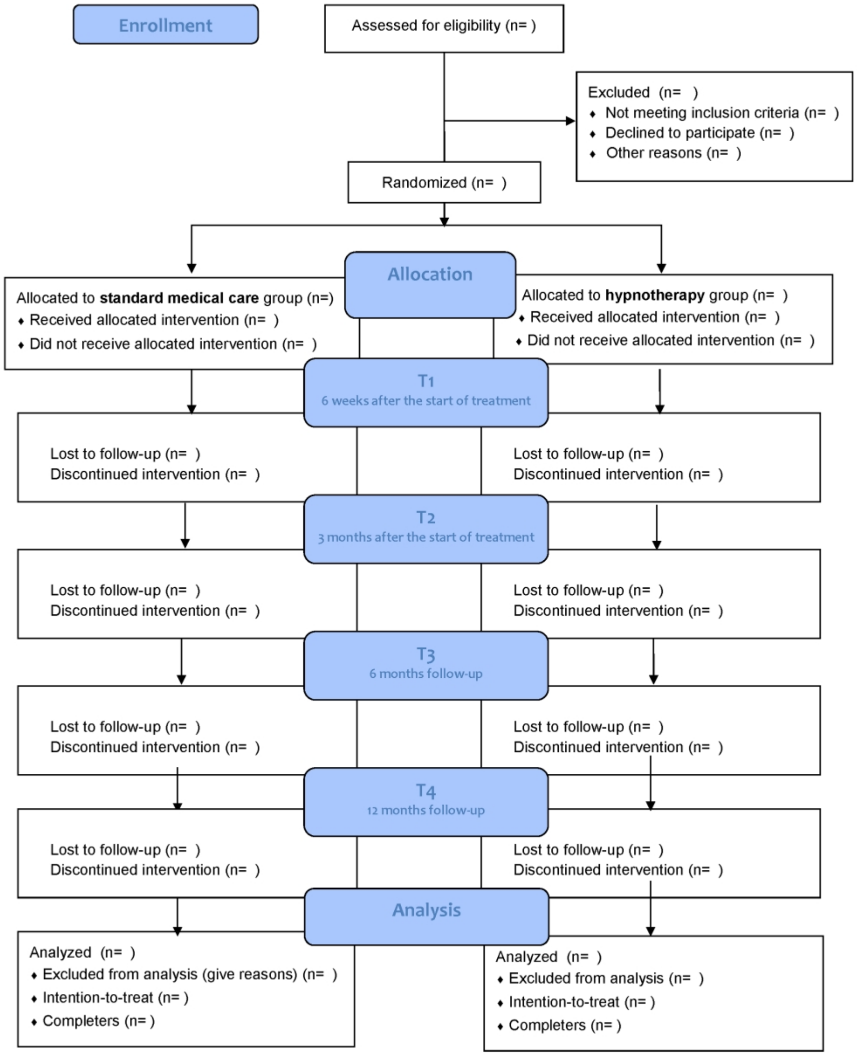

Figure 2. The Consolidated Standards of Reporting Trials (CONSORT) flow diagram indicating the number of participants throughout the study. After eligibility screening, children are randomized in either the hypnotherapy (HT) or standard medical care (SMT) group. Follow-up measurements take place at 6 weeks after the start of treatment (t1), 3 months after the start of treatment (t2), and 6 months follow-up (t3) and 12 months follow-up (t4).

215x279mm (300 x 300 DPI)

Reporting checklist for protocol of a clinical trial.

Based on the SPIRIT guidelines.

Instructions to authors

Complete this checklist by entering the page numbers from your manuscript where readers will find each of the items listed below.

Your article may not currently address all the items on the checklist. Please modify your text to include the missing information. If you are certain that an item does not apply, please write "n/a" and provide a short explanation.

Upload your completed checklist as an extra file when you submit to a journal.

In your methods section, say that you used the SPIRIT reporting guidelines, and cite them as:

Chan A-W, Tetzlaff JM, Altman DG, Laupacis A, Gøtzsche PC, Krleža-Jerić K, Hróbjartsson A, Mann H, Dickersin K, Berlin J, Doré C, Parulekar W, Summerskill W, Groves T, Schulz K, Sox H, Rockhold FW, Rennie D, Moher D. SPIRIT 2013 Statement: Defining standard protocol items for clinical trials. Ann Intern Med. 2013;158(3):200-207

For peer review only

		Reporting Item	Page Number
Title	#1	Descriptive title identifying the study design, population, interventions, and, if applicable, trial acronym	1
Trial registration	#2a	Trial identifier and registry name. If not yet registered, name of intended registry	2
Trial registration: data set	#2b	All items from the World Health Organization Trial Registration Data Set	3-14
Protocol version	#3	Date and version identifier	1
Funding	#4	Sources and types of financial, material, and other support	18
Roles and responsibilities: contributorship	#5a	Names, affiliations, and roles of protocol contributors	1, 18
Roles and	#5b	Name and contact information for the trial sponsor	18

responsibilities: sponsor contact information			
Roles and responsibilities: sponsor and funder	#5c	Role of study sponsor and funders, if any, in study design; collection, management, analysis, and interpretation of data; writing of the report; and the decision to submit the report for publication, including whether they will have ultimate authority over any of these activities	18
Roles and responsibilities: committees	#5d	Composition, roles, and responsibilities of the coordinating centre, steering committee, endpoint adjudication committee, data management team, and other individuals or groups overseeing the trial, if applicable (see Item 21a for data monitoring committee)	NA
Background and rationale	#6a	Description of research question and justification for undertaking the trial, including summary of relevant studies (published and unpublished) examining benefits and harms for each intervention	3, 4
Background and rationale: choice of comparators	#6b	Explanation for choice of comparators	5
Objectives	#7	Specific objectives or hypotheses	4
Trial design	#8	Description of trial design including type of trial (eg, parallel group, crossover, factorial, single group), allocation ratio, and framework (eg, superiority, equivalence, non-inferiority, exploratory)	5
Study setting	#9	Description of study settings (eg, community clinic, academic hospital) and list of countries where data will be collected. Reference to where list of study sites can be obtained	5
Eligibility criteria	#10	Inclusion and exclusion criteria for participants. If applicable, eligibility criteria for study centres and individuals who will perform the interventions (eg, surgeons, psychotherapists)	6
Interventions:	#11a	Interventions for each group with sufficient detail to allow	7-9

For peer review only

description		replication, including how and when they will be administered	
Interventions: modifications	#11b	Criteria for discontinuing or modifying allocated interventions for a given trial participant (eg, drug dose change in response to harms, participant request, or improving / worsening disease)	9, 15
Interventions: adherance	#11c	Strategies to improve adherence to intervention protocols, and any procedures for monitoring adherence (eg, drug tablet return; laboratory tests)	9
Interventions: concomitant care	#11d	Relevant concomitant care and interventions that are permitted or prohibited during the trial	9
Outcomes	#12	Primary, secondary, and other outcomes, including the specific measurement variable (eg, systolic blood pressure), analysis metric (eg, change from baseline, final value, time to event), method of aggregation (eg, median, proportion), and time point for each outcome. Explanation of the clinical relevance of chosen efficacy and harm outcomes is strongly recommended	9-13
Participant timeline	#13	Time schedule of enrolment, interventions (including any run-ins and washouts), assessments, and visits for participants. A schematic diagram is highly recommended (see Figure)	5
Sample size	#14	Estimated number of participants needed to achieve study objectives and how it was determined, including clinical and statistical assumptions supporting any sample size calculations	13-14
Recruitment	#15	Strategies for achieving adequate participant enrolment to reach target sample size	5
Allocation: sequence generation	#16a	Method of generating the allocation sequence (eg, computer-generated random numbers), and list of any factors for stratification. To reduce predictability of a random sequence, details of any planned restriction (eg, blocking) should be provided in a separate document that is unavailable to those who enrol participants or assign	6-7

For peer review only

		interventions	
Allocation concealment mechanism	#16b	Mechanism of implementing the allocation sequence (eg, central telephone; sequentially numbered, opaque, sealed envelopes), describing any steps to conceal the sequence until interventions are assigned	6-7
Allocation: implementation	#16c	Who will generate the allocation sequence, who will enrol participants, and who will assign participants to interventions	6-7
Blinding (masking)	#17a	Who will be blinded after assignment to interventions (eg, trial participants, care providers, outcome assessors, data analysts), and how	6-7
Blinding (masking): emergency unblinding	#17b	If blinded, circumstances under which unblinding is permissible, and procedure for revealing a participant's allocated intervention during the trial	NA
Data collection plan	#18a	Plans for assessment and collection of outcome, baseline, and other trial data, including any related processes to promote data quality (eg, duplicate measurements, training of assessors) and a description of study instruments (eg, questionnaires, laboratory tests) along with their reliability and validity, if known. Reference to where data collection forms can be found, if not in the protocol	9-13
Data collection plan: retention	#18b	Plans to promote participant retention and complete follow-up, including list of any outcome data to be collected for participants who discontinue or deviate from intervention protocols	9
Data management	#19	Plans for data entry, coding, security, and storage, including any related processes to promote data quality (eg, double data entry; range checks for data values). Reference to where details of data management procedures can be found, if not in the protocol	15
Statistics: outcomes	#20a	Statistical methods for analysing primary and secondary outcomes. Reference to where other details of the statistical analysis plan can be found, if not in the protocol	14-15

For peer review only

Statistics: additional analyses	#20b	Methods for any additional analyses (eg, subgroup and adjusted analyses)	NA
Statistics: analysis population and missing data	#20c	Definition of analysis population relating to protocol non-adherence (eg, as randomised analysis), and any statistical methods to handle missing data (eg, multiple imputation)	14
Data monitoring: formal committee	#21a	Composition of data monitoring committee (DMC); summary of its role and reporting structure; statement of whether it is independent from the sponsor and competing interests; and reference to where further details about its charter can be found, if not in the protocol. Alternatively, an explanation of why a DMC is not needed	15
Data monitoring: interim analysis	#21b	Description of any interim analyses and stopping guidelines, including who will have access to these interim results and make the final decision to terminate the trial	NA
Harms	#22	Plans for collecting, assessing, reporting, and managing solicited and spontaneously reported adverse events and other unintended effects of trial interventions or trial conduct	15
Auditing	#23	Frequency and procedures for auditing trial conduct, if any, and whether the process will be independent from investigators and the sponsor	15
Research ethics approval	#24	Plans for seeking research ethics committee / institutional review board (REC / IRB) approval	P1
Protocol amendments	#25	Plans for communicating important protocol modifications (eg, changes to eligibility criteria, outcomes, analyses) to relevant parties (eg, investigators, REC / IRBs, trial participants, trial registries, journals, regulators)	14
Consent or assent	#26a	Who will obtain informed consent or assent from potential trial participants or authorised surrogates, and how (see Item 32)	19
Consent or assent: ancillary studies	#26b	Additional consent provisions for collection and use of participant data and biological specimens in ancillary studies, if applicable	NA

For peer review only

Confidentiality	#27	How personal information about potential and enrolled participants will be collected, shared, and maintained in order to protect confidentiality before, during, and after the trial	15
Declaration of interests	#28	Financial and other competing interests for principal investigators for the overall trial and each study site	18
Data access	#29	Statement of who will have access to the final trial dataset, and disclosure of contractual agreements that limit such access for investigators	15
Ancillary and post trial care	#30	Provisions, if any, for ancillary and post-trial care, and for compensation to those who suffer harm from trial participation	15
Dissemination policy: trial results	#31a	Plans for investigators and sponsor to communicate trial results to participants, healthcare professionals, the public, and other relevant groups (eg, via publication, reporting in results databases, or other data sharing arrangements), including any publication restrictions	16
Dissemination policy: authorship	#31b	Authorship eligibility guidelines and any intended use of professional writers	NA
Dissemination policy: reproducible research	#31c	Plans, if any, for granting public access to the full protocol, participant-level dataset, and statistical code	NA
Informed consent materials	#32	Model consent form and other related documentation given to participants and authorised surrogates	NA
Biological specimens	#33	Plans for collection, laboratory evaluation, and storage of biological specimens for genetic or molecular analysis in the current trial and for future use in ancillary studies, if applicable	NA

The SPIRIT checklist is distributed under the terms of the Creative Commons Attribution License CC-BY-ND 3.0. This checklist was completed on 19. June 2018 using http://www.goodreports.org/, a tool made by the EQUATOR Network in collaboration with Penelope.ai

For peer review only

Gut-Directed Hypnotherapy Versus Standard Medical Care for Nausea in Children with Functional Nausea or Functional Dyspepsia: Protocol of a Multi-Center Randomized Trial.

Journal:	*BMJ Open*
Manuscript ID	bmjopen-2018-024903.R1
Article Type:	Protocol
Date Submitted by the Author:	21-Nov-2018
Complete List of Authors:	Browne, Pamela; Amsterdam UMC, Department of Pediatric Gastroenterology and Nutrition<br>den Hollander, Bibiche; Amsterdam UMC, Department of Pediatric Gastroenterology and Nutrition<br>Speksnijder, Esther; Amsterdam UMC, Department of Pediatric Gastroenterology and Nutrition<br>van Wering, Herbert; Amphia Ziekenhuis, Department of Pediatrics<br>Tjon a ten, Walther; Maxima Medical Center, Department of Pediatrics<br>George, Elvira; Northwest Clinics, Department of Pediatrics<br>Groeneweg, Michael; Maasstad Ziekenhuis, Department of Pediatrics<br>Bevers, Nanja; Zuyderland Medical Center, Department of Pediatrics<br>Wessels, Margreet; Rijnstate Hospital, Department of Pediatrics<br>van den Berg, Maartje; Haaglanden Medical Center, Department of Pediatrics<br>Goede, Joery; Spaarne Hospital, Department of Pediatrics<br>Teklenburg, Sarah; Isala Clinics, Department of Pediatrics<br>Frankenhuis, Carla; Amsterdam UMC, Department of Pediatric Gastroenterology and Nutrition<br>Benninga, Marc; Amsterdam UMC, Department of Pediatric Gastroenterology and Nutrition<br>Vlieger, Arine; St. Antonius Hospital, Department of Pediatrics
**Primary Subject Heading**:	Paediatrics
Secondary Subject Heading:	Gastroenterology and hepatology
Keywords:	Hypnotherapy, Functional Nausea, Functional Dyspepsia, Children, Adolescents

SCHOLARONE™
Manuscripts

21 November 2019. V2.

Gut-Directed Hypnotherapy Versus Standard Medical Care for Nausea in Children with Functional Nausea or Functional Dyspepsia: Protocol of a Multi-Center Randomized Trial.

Author names and affiliations

Pamela D. Browne[1], Bibiche den Hollander[1], Esther Speksnijder[1], Herbert van Wering[2], Walther Tjon A Ten[3], Elvira K. George[4], Michael Groeneweg[5], Nanja Bevers[6], Margreet Wessels[7], Maartje van den Berg[8], Joery Goede[9], Sarah Teklenburg[10], Carla Frankenhuis[1], Marc A. Benninga[1], Arine M. Vlieger[11]

Corresponding author

Pamela D. Browne; P.D.Browne@amc.uva.nl

Present/permanent address: Emma Children's Hospital, Amsterdam UMC, University of Amsterdam, Pediatric Gastroenterology, Meibergdreef 9 (room C2-312), 1105 AZ Amsterdam, The Netherlands

1. Emma Children's Hospital, Amsterdam UMC, University of Amsterdam, Pediatric Gastroenterology, Amsterdam, The Netherlands
2. Department of Pediatrics, Amphia Hospital, Breda, the Netherlands
3. Department of Pediatrics, Maxima Medical Center, Veldhoven, the Netherlands
4. Department of Pediatrics, Northwest Clinics, Alkmaar, The Netherlands
5. Department of Pediatrics, Maasstad Hospital, Rotterdam, The Netherlands
6. Department of Pediatrics, Zuyderland Medical Center, Heerlen, The Netherlands
7. Department of Pediatrics, Rijnstate Hospital, Arnhem, The Netherlands
8. Department of Pediatrics, Haaglanden Medical Center, Den Haag, The Netherlands
9. Department of Pediatrics, Spaarne Hospital, Hoofddorp, The Netherlands
10. Department of Pediatrics, Isala Clinics, Zwolle, The Netherlands
11. Department of Pediatrics, St. Antonius Hospital, Nieuwegein, The Netherlands

For peer review only

For peer review only - http://bmjopen.bmj.com/site/about/guidelines.xhtml

21 November 2019. V2.

Abstract

Introduction: The treatment of chronic functional nausea or nausea due to functional dyspepsia in children is generally symptomatic. Moreover, these disorders pose a risk for worse psychosocial and health outcomes in children. Hypnotherapy, by its ability to positively influence gastrointestinal and psychosocial functioning, may be an effective treatment for chronic nausea.

Methods and analysis: To test efficacy, this multi-center, parallel, randomized controlled, open label trial evaluates whether gut-directed hypnotherapy (HT) is superior to standard medical care (SMT) for reducing nausea. The study will be conducted at eleven academic and non-academic hospitals across the Netherlands. A total of hundred children (8-18 years), fulfilling the Rome IV criteria for chronic idiopathic nausea or functional dyspepsia with prominent nausea, will be randomly allocated (1:1) to receive HT or SMT. Children allocated to the HT group will receive six sessions of HT during three months, while children allocated to the SMT group will receive six sessions of SMT + supportive therapy during the same period. The primary outcome will be the difference in the proportion of children with at least 50% reduction of nausea, compared to baseline at twelve months follow-up. Secondary outcomes include the changes in abdominal pain, dyspeptic symptoms, quality of life, anxiety, depression, school absences, parental absence of work, health-care costs, and adequate relief of symptoms, measured directly after treatment, six and twelve months follow-up. If HT proves effective for reducing nausea, it may become a new treatment strategy to treat children with chronic functional nausea or functional dyspepsia with prominent nausea.

Ethics and dissemination: Results of the study will be publically disclosed to the public, without any restrictions, in peer-reviewed journal and international conferences. The study is approved by the Medical Research Ethics Committees United (MEC-U) in the Netherlands.

Registration details: Dutch trial registration number is NTR5814. Registered on 7 June 2016.

http://www.trialregister.nl/trialreg/admin/rctview.asp?TC=5814

Keywords: Hypnotherapy, Functional Nausea, Functional Dyspepsia, Children, Adolescents

Words: 5972

For peer review only - http://bmjopen.bmj.com/site/about/guidelines.xhtml

21 November 2019. V2.

Article summary (strengths and limitations)

- First study to investigate the effectiveness of hypnotherapy on symptoms of nausea in children and adolescents, diagnosed with chronic idiopathic nausea or functional dyspepsia.
- A multicenter study with eleven participating academic and non-academic hospitals recruiting 100 children and adolescents.
- Long-term study follow-up of one year.
- Due to the nature of hypnotherapy, children, parents, and health care providers are not blinded for the received treatments.

1. Introduction

Chronic idiopathic nausea (CIN) and functional dyspepsia (FD) affect approximately 0.5% and 4.5-7.6% children worldwide [1], respectively, and are associated with substantial physical and psychosocial distress, school absences and decreased social functioning [2-4]. Moreover, it has a considerable negative financial impact on health care [5]. According to the Rome IV criteria, when no evidence of organic disease is found, the disorders are considered functional. Children meet the Rome IV criteria for CIN when they suffer from chronic nausea without abdominal pain, when symptoms are not related to meals, and not consistently associated with vomiting. Children are diagnosed with FD when they have chronic symptoms of epigastric pain/burning, symptoms of postprandial fullness, and/or early satiation [6].

The treatment of CIN and FD with prominent nausea in pediatric patients is mostly symptomatic and not well defined. Most clinicians individualize the patient's medical treatment, including prokinetics, anti-emetics, antacids, and herbal products, according to their symptoms and associated comorbidities [3, 4]. The major disadvantage of this approach is that this treatment is symptomatic, and thus drugs often need to be used as long as patients suffer from nausea, which may take years [7, 8]. Hence, there is a need for additional effective treatments for nausea in children with CIN or FD.

Several pathophysiologic mechanisms have been proposed to play a role in the etiology of CIN and FD, including delayed gastric emptying, impaired gastric motility and/or abnormal central nervous system processing of gastric stimuli through the gut-brain axis [3]. Additionally, there are indications that psychological factors, including anxiety and stress, may increase the severity of nausea through the gut-brain axis [9, 10].

Gut-directed hypnotherapy (HT) may have the potential to reduce symptoms of

For peer review only

nausea in children with CIN or FD. HT is a form of therapy in which a therapist, by using suggestions, can induce a hypnotic state in an individual to positively modify physiological, cognitive, and affective processes, as well as behavior in that individual [11]. It has been shown to be very effective in the treatment of adults and children with functional abdominal pain [12, 13] and children with chemotherapy-induced nausea and vomiting [14]. Therefore we hypothesize that HT, by its ability to influence gut motility [15], psychological well-being [16] and visceral hypersensitivity [17-19], might alleviate symptoms of nausea in children with CIN or FD as well. To date, however, no studies have examined the potential effect of HT in children with CIN or FD.

The main goal of this multi-center randomized controlled trial is to evaluate the effectiveness of HT in reducing symptoms of nausea in children with CIN or FD. Six sessions of gut-direct HT will be compared with six sessions of SMT plus supportive therapy in 100 children with CIN or FD between 8 and 18 years. Additionally, we will investigate the potential influence on abdominal pain, dyspeptic symptoms, quality of life, anxiety, depression, school absences, parental absence of work and health-care costs. We expect that HT will be more effective in reducing symptoms of nausea than standard medical care. Furthermore, we expect that children receiving hypnotherapy will report more relief of symptoms (e.g. less abdominal pain, less dyspeptic symptoms), better quality of life, less symptoms of anxiety and depression, less absence from school, compared to children receiving standard medical care. We also expect that parents of children in the hypnotherapy group will report less parental absences from work and lower health care costs, compared the medical care group.

For peer review only

21 November 2019. V2.

2. Methods

2.1 Trial design

The present study in children and adolescents is a multi-center randomized controlled trial (RCT). Hundred children between ages 8 and 18 years with symptoms of nausea and fulfilling the Rome IV criteria for CIN or FD, diagnosed by their pediatrician, will be enrolled in the study. After randomization, children will receive either 6 sessions of gut-directed HT during 3 months by a qualified hypnotherapist, or 6 sessions of SMT plus supportive therapy from their pediatrician during 3 months (see Figure 1). Detailed information on the HT and SMT interventions can be found under the section '2.5. Intervention'. The additional file 1 presents the Standard Protocol Items: Recommendations for Interventional Trials (SPIRIT) checklist (see additional File 1).

2.2. Patient and Public Involvement

Patients and the public were not involved in the design of the RCT.

2.3 Recruitment

2.3.1 Recruitment procedures

Children and adolescents will be recruited in outpatient pediatric clinics of 1 academic and 10 non-academic hospitals in the Netherlands: Academic Medical Center (Amsterdam), Amphia Hospital (Breda), Maxima Medical Center (Veldhoven), Northwest Clinics (Alkmaar), Maasstad Hospital (Rotterdam), Zuyderland Medical Center (Heerlen), Rijnstate Hospital, (Arnhem), Haaglanden Medical Center (Den Haag), Spaarne Hospital (Hoofddorp), Isala Clinics (Zwolle) and St. Antonius Hospital (Nieuwegein). These centers are located in both urban and rural areas throughout the Netherlands, serving an ethnically diverse pediatric population.

2.3.2. Participant screening

All children with symptoms of nausea and fulfilling the Rome IV criteria for CIN or FD, will undergo blood laboratory testing before inclusion, including complete blood cell count, C-reactive protein, liver function tests, creatinine, total bilirubin, and for celiac screening, amylase anti-transglutaminase antibodies and IgA testing. Additionally, urinalysis and stool analysis for parasites (*Giardia Lamblia, Entamoeba Histolytica*) and *Helicobacter pylori* antigens will be performed. The need for additional diagnostic testing, for example

For peer review only

endoscopy to rule out eosinophilic esophagitis or 24h pH, will be left to the discretion of the treating pediatrician or pediatric gastroenterologist. The flow of the study protocol is presented in Figure 2.

2.4. Criteria

2.4.1. Inclusion criteria

A total sample of 100 children and adolescents with CIN or FD with symptoms of nausea will be enrolled in the study. Children and adolescents can participate in this study if they meet the following inclusion criteria:

- Age 8 to 18 years at inclusion of the study
- Diagnosis of CIN or FD, with symptoms of nausea, according to Rome IV criteria (Hyams et al., 2016)
- Sufficient knowledge of the Dutch language

2.4.2. Exclusion criteria

Children will not be enrolled in the study if they meet the following exclusion criteria:

- Concomitant organic gastrointestinal disease
- Simultaneous treatment by another health care professional for symptoms of nausea
- Previously received hypnotherapy
- Intellectual disability

2.5. Randomization, blinding, and treatment allocation

After obtaining informed consent, children are randomly allocated, by the treating pediatricians, to one of the two treatment arms: HT, given by a qualified therapist, or SMT, meaning treatment by the child's pediatrician. A computerized random-number generator will be used to randomly allocate children on a 1:1 basis with varying block sized of 2, 4 and 6. To ensure allocation concealment, central randomization will be applied and the random allocation sequence remains concealed from pediatricians enrolling children into the study. Due to the nature of HT, it is not possible to blind the participating children and health care professionals involved in the treatment of the participants.

2.6. Intervention

2.6.1. Hypnotherapy

For peer review only - http://bmjopen.bmj.com/site/about/guidelines.xhtml

Individual HT consist of 6 sessions of 50-60 minutes, given over a period of 3 months by a qualified hypnotherapist (week 1, 2, 3, 5, 7 and 11). Twelve hypnotherapist affiliated to the recruiting hospitals will offer the HT to children. All hypnotherapists have years of experience in performing HT in children. The hypnotherapists will use an adapted version of our previously used HT protocol [13, 20]. The HT protocol contains exercises focusing on normalization of the gut motility, stress reduction and ego strengthening. The hypnotherapists will be instructed to use the same scripts, but are allowed to adapt the content to the child's needs. The same protocol is used for children of all ages. However, the language used will be adjusted to the child's developmental age.

In the first session, an introduction to HT will be given to the child and parents, including an explanation of what HT is and how it may help in reducing symptoms of nausea. Furthermore, the hypnotherapist will take a full history and children and parents are instructed to not talk about the nausea during the treatment period. The hypnotherapist will then start with a breathing exercise and introduce a progressive relaxation, in which children imagine floating on a big cloud. Positive suggestions to increase the child's belly comfort will also be provided. For instance, the child will be instructed to make hands warm and place both hands on the belly, imagining warmth spreading through their abdomen, and especially the stomach.

In the second session, the therapist will repeat the exercise on progressive relaxation. Additionally, the therapist will introduce an exercise focusing on reduction of anxiety and stress, which is called 'the favorite place exercise'.

The third session focuses on ego strengthening and a new exercise will be introduced: 'the rainbow planet exercise' for children attending primary school, and 'the air balloon exercise' for children in secondary school. In the first exercise, children choose a personal need from a rainbow that contains different needs, for example a healthy stomach, courage, tranquility, or confidence.

In the fourth session, children are encouraged to release stress during the 'the beach without worries exercise' and additional ego strengthening suggestions are made.

The fifth session focuses on reduction of anxiety, stress and ego strengthening, as well as improved functioning of the digestive system. For the digestive system, children visualize a well working digestive system with food sliding through the stomach and bowel in a comfortable way.

For peer review only - http://bmjopen.bmj.com/site/about/guidelines.xhtml

21 November 2019. V2.

In the sixth session, the previous sessions will be evaluated, remaining gastro-intestinal problems may be addressed and preceding exercises may be repeated, if requested by children. If no improvement has taken place, an exercise will be introduced in which the child is instructed to look inside the stomach to see 'what the stomach needs'.

After the first session, all children will receive a CD containing standard scripts of the exercises used during the sessions. The hypnotherapist will advise children to self-practice these exercises on a daily basis. Additionally, the therapist will encourage children to practice breathing exercises a few time a day.

2.6.2. Standard medical care + supportive therapy

In the SMT group, children will visit their treating pediatrician 6 times over a 3-month period. All pediatricians will be instructed to use the same protocol for treating symptoms of nausea. The protocol consists of a stepwise approach. In the first step, children and parents will be educated about CIN and FD, reassured that there is no structural organic underlying disease present, and dietary and lifestyle advices will be provided. Children will be advised to adhere to national practical guidelines for healthy eating by the Netherlands Nutrition Centre. Children will be recommended to avoid products containing caffeine, strong spices, citrus fruits, onions, fatty foods, and if applicable, to stop smoking. Additionally, the pediatrician will explore, together with children and parents, possible connections between stressful moments, emotional problems and complaints of nausea. If connections are present, the pediatrician will encourage children and parents to improve coping strategies to effectively manage stress, to reduce external stressors, and to ensure an optimal environment with sufficient relaxation. Children will also be encouraged to continue their normal daily and sport activities and to go to school, to prevent or decrease avoidance behavior.

In case this does not result in adequate relief of symptoms, the pediatrician continues with the second step. In the second step, proton pump inhibitors (PPI) in combination with domperidone will be prescribed. If this treatment is not effective in reducing symptoms the pediatrician continues with the third step, which includes the prescription of ondansetron and (dis)continuation of PPIs. If again no adequate improvement occurs, in the fourth step, Iberogast will be started for children >12 years. If children do not respond to Iberogast (>12 years) or ondansetron (<12 years), the pediatrician will continue with the fifth step and

For peer review only - http://bmjopen.bmj.com/site/about/guidelines.xhtml

21 November 2019. V2.

prescribe erythromycin. Finally, if previous treatment did not prove to be successful in reducing symptoms, cyproheptadine will be prescribed.

The pediatrician will evaluate each step after two to four weeks. All dosages, except for Iberogast, will be prescribed according to www.kinderformularium.nl (Dutch medical guideline for pediatricians).

In addition to the medical treatment, children will receive 6 half hour sessions of supportive therapy given by the treating pediatrician. In these sessions, the symptom progression will be discussed and patient education will be provided. Moreover, exploration of potential contributing triggers (i.e. dietary product, emotional problems and stressful events) will be evaluated together with children and parents. Supportive therapy is added to correct for the patient-therapist time in the HT group.

2.6.3. Co-interventions

After 6 sessions of HT, children visit their pediatrician to evaluate the effects of HT and, if considered necessary, to receive additional medical care.

2.7. Outcomes

2.7.1. Primary outcome

The primary outcome of the RCT is the proportion of patients with at least 50% reduction of their symptoms of nausea compare to baseline at 12 months follow-up. Children and adolescents report symptoms of nausea at home by using the 7-day diary. To promote retention and complete follow-up, children and parents will be reminded on regular basis, via email and phone calls, to fill in the 7-day diary and other questionnaires.

7-day diary

The 7-day diary is used by children and adolescents to score the severity, incidence and frequency of symptoms of nausea, every day during 7 consecutive days [13, 21, 22].

The *severity* of nausea is assessed by the ‘nausea face’ analog scale, a validated tool in the pediatric population [23]. Children rate their degree of nausea on a day using 6-faces: face 0 indicates no nausea and face 6 indicates nausea as bad as it can be imaged. Scores on the ‘nausea face’ scale are transported to a daily 0-10 score. Face 0, no nausea, is scored as 0, face 1 is scored as 2, face 2 is scored as 4, face 3 is scored as 6, face 4 is scored as 8 and face

For peer review only - http://bmjopen.bmj.com/site/about/guidelines.xhtml

5 is scored as 10 [23]. The 'severity of nausea' (SON) score is calculated by summing up the scores of seven days, giving a maximum of score of 70 [13, 20- 22].

The *incidence* of nausea is assessed using the 'nausea incidence' scale (NIS), adapted from the 5-point dyspepsia Likert scale (Canan, 2011; De Luca et al., 2004). It measures the incidence of symptoms during a day, where score 0 for no nausea, score 1 for 1-2 times a day, score 2 for 3-5 times a day, score 3 for intermittent complaints and score 4 for complaints were always present. The total sum of the scores of seven days indicates the severity of the nausea during a week, as experienced by the child. The maximum total score is 28 [13, 20- 22].

The *frequency* of symptoms of nausea is recorded in minutes/hours per day and is scored by children as 0 when there was no nausea, 1 if children had <10 minutes of nausea, 2 for 10-30 minutes of nausea, 3 for 30 minutes-2 hours of nausea, 4 for 2-4 hours of nausea and 5 if the nausea lasts >4 hours a day. The 'nausea frequency score' (NFS) is calculated by summing the scores of the seven days, with a maximum of 35 [13, 21, 22]. Treatment success is defined as at least 50% reduction in the SON, NIS and NFS.

2.7.2. Secondary outcomes

In addition to the primary outcomes, the present study investigates secondary outcomes, including abdominal pain, dyspeptic symptoms, health related quality of life, anxiety, depression, school absences, parental absence of work and health-care costs. Secondary outcomes are measured at 6 weeks and 3 months after treatment, and at 6 and 12 months follow-up after the end of treatment (Figure 1).

Abdominal pain

A 7-day diary is used to assess the severity and frequency of abdominal pain, every day during 7 consecutive days. It comprises of two subscales: the abdominal pain intensity subscale (APIS) and abdominal pain frequency subscale (APFS). The APIS will be scored using an affective facial scale ranging with face 0 indicating 'no pain at all' to face 5 indicating 'the most severe pain'. No abdominal pain is scored as 0, faces 1 - 2 are scored as 1, faces 3 - 4 are scored as 2 and face 5 is scored as 3. The scores of seven days are summed up, with a maximum score of 21 [13, 20- 22]. The APFS is recorded in minutes/hours of abdominal pain per day, with score 0 indicating no pain, score 1 if children had <10 minutes of pain, 2 for 10-

For peer review only - http://bmjopen.bmj.com/site/about/guidelines.xhtml

30 minutes of pain, 3 for 30 minutes-2 hours of pain, 4 for 2-4 hours of pain and 5 >4 hours of pain. The scores the APFS are summed up, giving a pain frequency score of maximum 35 [13, 20- 22].

Dyspeptic symptoms

Severity and incidence of dyspeptic symptoms is measured using the 5-point dyspepsia Likert scale, previously used in the pediatric population [24, 25]. The dyspepsia Likert scale consists of 8 gastrointestinal dyspeptic symptoms: epigastric pain, upper abdominal discomfort, retrosternal pyrosis, sour-bitter taste, halitosis, belching, nausea, and early satiety. Children score the *severity* of each symptoms during the previous two weeks on a 5-point Likert scale: score 1 'no complaints at all', score 2 'little complaints', score 3 'moderate complaints', score 4 'quite a lot of complaints' and 5 'serious complaints' [24]. A higher sum score indicates more severe dyspeptic complaints (SDC score), with a maximum of 40. Children report on the *incidence* of each of the symptoms during the previous two weeks by scoring: 1 'no complaints', 2 '1-2 times a week', 3 '3-5 times a week', 4 'intermittent complaints' and 5 'complaints were always present' [24]. The dyspepsia severity score (DSS) is calculated by summing up the scores, giving a maximum value of 40.

Health related quality of life

The KIDSCREEN-52 questionnaire measures health related quality of life (QoL) in children and adolescents. The questionnaire has been shown a valid tool in the Dutch pediatric population [26, 27]. The KIDSCREEN-52 consists of items on ten dimensions related to QoL on a 5-point Likert scale: moods and emotions, self-perception, relations with parents and home life, autonomy, physical well-being, psychological well-being, school environment, social support and peers, social acceptance (bullying) and financial resources. For each individual dimension, Rasch scores are computed from the individual items. These are then transformed into T-values: higher T-values indicate a better health related QoL and well-being.

Anxiety and depression

Anxiety and depression are evaluated using the Revised Anxiety and Depression Scale-short version (RCADS-25). This questionnaire has been previously validated in the Dutch pediatric population [28]. The RCADS-25 consists of five subscales measuring symptoms of generalized anxiety disorders, separation anxiety disorder, social phobia, panic disorder and major depressive disorder. Each subscale contains five items and scales range from 0 (never) to 3

For peer review only

(always). The total score on anxiety or depression is the sum of the items measuring symptoms of anxiety and depressive symptoms, respectively. Higher scores indicate more symptoms of anxiety or depression.

Cost effectiveness/cost utility

The Health Utility Index Mark 3 (HUI) will be used in the cost-utility and cost-effectiveness analysis. The HUI-3 is a multi-attribute utility measure of health status in children as reported by parents. Proxy measurements of parents for health status of children are justifiable, as some children may be too young to provide reliable and valid information about their own health status [29-31]. The questionnaire consists of eight dimensions of health status: vision, hearing, speech, ambulation, dexterity, emotion, cognition, and pain, with scales varying from highly impaired to normal. Health utilities of 1 indicate perfect health, whereas 0 indicates death. The Quality adjusted life years (QALY) will be calculated by multiplying the sum of the utility of health states by the time in between measurements.

Work absenteeism by parents and school absenteeism by children

An adapted version of the Dutch Health and Labor Questionnaire (HLQ) will be used to measure work absenteeism by parents, school absenteeism by children, and indirect costs of health care utilization [32]. This adapted version contains three items. Parents indicate whether their child has been absent from school due to complaints of nausea, and if yes, the amount of hours per week. For work absenteeism by parents, parents indicate the number of hours they worked less on average because of their child's symptoms of nausea. For the indirect costs of health care utilization, parents indicate additional costs they had due to symptoms of nausea of their child over the past four weeks.

Somatization

The Children's Somatization Inventory (CSI) measures the extent to which children and adolescents experience somatic symptoms. The questionnaire has been shown a valid and reliable self-report instrument in the pediatric population [33]. The CSI consists of 35 items and on a 5-point Likert scale (0 = not at all to 4 = a whole lot) and children rate the extent to which they experienced somatic symptoms in the previous two weeks. The total score is calculated by summing up the 35 items, with higher scores indicating higher intensity of somatic complaints experienced by the child.

In order to calculate a separate CSI-score for non-gastrointestinal (GI) symptoms, 7 items on GI-symptoms (nausea, constipation, diarrhea, epigastric and abdominal pain, vomiting and

For peer review only - http://bmjopen.bmj.com/site/about/guidelines.xhtml

bloating) are left out. The total score of somatic symptoms without GI-symptoms is calculated by summing up the scores of non-GI symptoms, with higher scores reflecting higher intensity of non-GI somatic complaints.

Adequate relief

Parents and children will be asked whether adequate relief of symptoms of nausea has occurred, using a dichotomous scale (yes/no). Adequate relief has been previously used as an endpoint in clinical trials assessing hypnotherapy in children and adolescents [20] and has been shown a valid outcome measure for functional gastrointestinal disorders [34].

2.8. Sample size calculation

The primary outcome of the RCT is the proportion of patients with at least 50% reduction of their symptoms of nausea compare to baseline at 12 months follow-up. Based on our pilot study (Vlieger, A. M. "A pilot-study of hypnotherapy as a treatment for functional nausea in children") and the success percentages in studies using hypnotherapy in adults with functional dyspepsia [35] and in pediatric cancer patients [14] , we expect that 80% of the children in the hypnotherapy group will have >50% reduction of their symptoms of nausea after one year. In the standard medical care group, we anticipate that 50% of the children will have >50% reduction of their symptoms of nausea after one year. Based on these expected proportions, 45 children per group will be needed to achieve a power of 80% with a one-sided significance level of 5%. Accounting for a 10% dropout, 100 children will be included in this study. If a child is prematurely withdrawn from the study, he/she will not be replaced; data will be analyzed according to the intention to treat analysis.

2.9. Statistical analysis

2.9.1. Primary outcome

Outcomes will be analyzed according to the intention to treat (ITT) analysis. For the primary outcome, the Chi-square test will be used to compare the proportions of patients with >50% reduction of symptoms of nausea (i.e. severity, incidence and frequency of nausea) after 12 months follow-up between the two groups (HT versus SMT). For all analysis, the significance level for statistical analysis is set at $\alpha = 0.05$. Multiple imputation will be applied to deal with cases of missing data.

For peer review only - http://bmjopen.bmj.com/site/about/guidelines.xhtml

21 November 2019. V2.

2.9.2. Secondary outcomes

For the secondary outcomes, including the potential influence on abdominal pain, dyspeptic symptoms, health related quality of life, anxiety, depression, work absenteeism by parents, school absenteeism by children, somatization and adequate relief, the Student's *t*-test will be used for means of normally distributed data, the Mann–Whitney U test for nonparametric data and the Chi-square test to compare proportions. To calculate the cost-effectiveness, cost-utility and cost-effectiveness ratios will be calculated for the extra costs per child with >50% reduction of symptoms of nausea and the extra costs per QALY.

As secondary analysis, the proportion of patients with >50% reduction of their nausea (severity, incidence and frequency) after treatment, 6 months and 12 months follow up will be compared between groups using multivariate logistic regression correcting for age and center.

3. Amendments

Prior to implementation, amendments will be examined and approved by the METC. The sponsor will only record non-substantial amendments.

4. Data monitoring

Hypnotherapy is usually well tolerated in children, without significant side effects. In our previous trials only a minority of children reported some dizziness, mostly during or directly after the end of the first session [13, 21]. In case children experience dizziness, they will be advised to execute the remaining sessions in a sitting position instead of a supine position. Children assigned to the SMT group will receive standard medical treatment, including drugs that have been either registered for children, or of which side effects are limited and well known. For these reasons, no Data Monitoring Safety Board will be established.

Study auditing will be accomplished by periodic visits to the participating centers, and by email and telephone contact with local investigators, to ensure the study protocol is being complied with and to discuss any problems that might have arisen.

5. Potential harms

In accordance to the legal requirements in the Netherlands (article 10, subsection 1, WMO), the investigator will inform the subjects and the reviewing accredited METC if harmful events occur. When there are indications that the disadvantage of participation may be

For peer review only - http://bmjopen.bmj.com/site/about/guidelines.xhtml

significantly greater than was described in the research proposal, the study will be suspended pending further review by the accredited METC. However, the study will not be suspended if it would jeopardize participating children's health.

6. Ancillary and post-trial care

In accordance with Article 7 WMO and the Measure regarding Compulsory Insurance for Clinical Research in Humans of 23th June 200th, the sponsor has liability insurance for any damage to children which might emerge from study participation [36].

7. Data storage

The related information on paper, including 7-day diaries, will be securely stored in a locked file cabinet with limited access. Online questionnaire data will be securely stored using the University's password-protected access systems. Only the main researchers will be given full access to the questionnaire data. All records that contain names will be saved in one file, which will be password protected and only accessible to the main researchers.

8. Dissemination policy

The researchers will communicate trial results to the public, health care providers, and other relevant groups via reports, and by publishing in peer-reviewed journals. Negative as well as positive results will be published. The results will be shared with participating children and parents after completion of the trial. All authors who provided substantial contributions to the conduct, interpretation, and reporting of the results will be granted authorship on the final trial report.

9. Discussion and conclusion

Chronic nausea is a highly disabling symptom for children with CIN or FD, and poses a risk for negative health outcomes and decreased psychosocial functioning [2-4]. To date large randomized placebo controlled trials evaluating the effect of any drug in children with either CIN or FD are lacking [37]. Current medical treatment is experienced based, however these treatments are symptomatic and often used for months or years [7, 8]. For these reasons, new effective treatment options to reduce nausea in children with CIN or FD are warranted.

There are indications that HT can decrease symptoms of functional nausea and dyspepsia in

For peer review only

21 November 2019. V2.

adults [35], and functional abdominal pain (FAP) [13] and chemotherapy induced nausea in children [14]. Calvert et al. (2002) found that adult patients with FD receiving 12 sessions of HT had significantly less dyspeptic symptoms (59%, N=26) compared to patients receiving medical treatment (33%, N=29) (p=0.02) [35]. These beneficial effects were maintained for more than a year: 56 weeks after the first treatment, 73% of the patients in the HT group reported symptom improvement compared to 43% in the medical treatment group (p<0.01). In children with FAP, Vlieger (2007) found that HT was highly superior compared to standard medical care to reduce abdominal pain. At one year follow-up, 85% of the children in the HT group (N=26) were in clinical remission compared to 25% of the children in the SMT group (N=24) (p<0.001) [13]. Additionally, a systematic review including six RCTs evaluating the effectiveness of hypnotherapy to reduce chemotherapy-induced nausea found hypnotherapy was most effective when compared with standard medical care to reduce complaints (D=0.99) [14].

The present study is the first study to investigate the effectiveness of HT on symptoms of nausea in children and adolescents diagnosed with CIN or FD, according to the Rome IV criteria. If shown effective, it may provide an additional treatment option for children with CIN or FD.

The study has several strengths. The first strength is that pediatricians from eleven different hospitals throughout the Netherlands will recruit all children and adolescents. The hospitals, both an academic center and teaching hospitals, serve an ethnic and socio-economic diverse population of children and adolescents. This recruitment method has two advantages: first, it may reduce response bias to the intervention. It has previously been reported that patients from primary and secondary level care may have different responses to treatment [38]. All children included in the present study receive secondary level care. The second advantage is that the multicenter design of the study will increase generalizability of the trial outcomes.

Another strength of the study is the long-term follow up of one year, which allows us to properly compare the potential effectiveness of HT with SMT. It has been known that the severity of functional gastrointestinal symptoms in children varies over time [39] and a continuous improvement in symptoms is often reported in children receiving hypnotherapy [13].

For peer review only - http://bmjopen.bmj.com/site/about/guidelines.xhtml

21 November 2019. V2.

502 This study also has several limitations. The first limitation is that children, parents, investigators and health care providers are not blinded for the received treatments, which is not possible due the nature of HT. Several solutions will be applied to limit the risk of bias. First, to minimize performance bias, pediatricians and hypnotherapists will follow treatment guidelines to prevent any use of additional or alternative forms of care during the study period that may influence treatment outcomes. Second, to reduce the risk of detection bias, children and adolescents use reliable outcome measures and record symptoms themselves at home. Moreover, children and adolescents record symptoms of nausea for seven consecutive days, which corrects for individual variability of symptoms over time. Third, the endpoints of the study will be preregistered.

512 The second limitation is that the SMT provided by pediatricians in this study may not reflect usual clinical practice. In the present study, children and adolescents receive longer and more intensive medical therapy from their pediatrician compared to the real medical practice situation. However, previous studies indicate that patient-provider interactions can largely influence gastrointestinal treatment outcomes [40]. Therefore, it is important to control for the time spent per patient in the SMT group.

518 If the results of this study show that HT given by a therapist is comparable or slightly more effective than medical treatment provided by pediatricians, HT may become a new treatment strategy to help children with CIN or FD. Furthermore, as HT is presumably less costly than treatments by a specialist it may also decrease health care costs.

522 **Abbreviations**

523 **APIS**: Abdominal Pain Intensity Subscale; **APFS:** abdominal pain frequency subscale**; CIN:** Chronic Idiopathic Nausea**; CSI:** Children's Somatization Inventory; **FAP**: Functional Abdominal Pain**; FD:** Functional Dyspepsia**; FGID:** Functional Gastrointestinal Disorders; **GI:** gastrointestinal; **HLQ**: Health and Labor Questionnaire; **HT:** Hypnotherapy; **HUI:** Health Utility Index Mark 3**; ITT:** Intention to Treat Analysis; **MEC-U:** Medical Research Ethics Committees United; **NIS**: Nausea Incidence Scale; **NFS:** Nausea Frequency Score; **PPI:** Proton Pump Inhibitors; **QALY:** Quality Adjusted Life Years**; QoL:** Health related Quality of Life**; RCADS-25:** Revised Anxiety and Depression Scale-short version**; RCT:** Randomized Controlled Trial; **REC**: Research Ethical Committees**; SMT:** Standard Medical Care plus supportive therapy; **SON**: Severity Of Nausea; **WMO**: Medical Research Involving Human Subjects Act

For peer review only - http://bmjopen.bmj.com/site/about/guidelines.xhtml

21 November 2019. V2.

Acknowledgements

None

Competing interests

The authors declare no competing interests.

Funding

The Christine Bader Foundation and Gastrointestinal and Liver Foundation supported this work. These funding sources had no role in the design of this study. They will not have any role during the collection of data, analyses or submission of the results.

Author contributions

AV is the principle investigator, designed the study, wrote the protocol, supervised the trial and supervised writing the manuscript. PB provided adjustments to the protocol, drafted the manuscript and coordinated the trial. MB critically revised the protocol, supervised the trial and supervised writing the manuscript. BdH, ES, HvW, WTAT, EK, MG, NB, MW, MvdB, JG, ST, CF participated in patient recruitment and/or treatment, read and approved the manuscript.

Trial status

Recruitment started in September 2016 and is ongoing. In October 2016, the first participant enrolled. Currently, 92 children are participating in the study (18 of them have completed the study protocol).

Ethics approval and consent to participate

This RCT was approved by the Medical Research Ethics Committees United (MEC-U) in Nieuwegein, the Netherlands (file number: NL51167.100.15). The study will be conducted in accordance with the Medical Research Involving Human Subjects Act (WMO) and conferring to the principles of the Declaration of Helsinki [36]. (64th WMA General Assembly, Fortaleza, Brazil, October 2013). The study will follow the conduct code concerning resistance in minors who participate in clinical trials as defined by the Dutch Pediatric Society. Informed consent will be asked from parents/guardians of children <12 years of age. In children and adolescents ≥12 years of age informed consent will be asked from the parents-guardians and the children and adolescents. In the event of amendments of the protocol, relevant

For peer review only

21 November 2019. V2.

research ethical committees (RECs) will be informed. Results of the study will be publically disclosed in a peer-reviewed journal, without any restrictions; both positive as well as negative results will be published.

5 peer reviewers suggested

Miguel Saps (msaps@childrensmemrial.org), Miranda van Tilburg (Tilburg@med.unc.edu), Peter Whorwell (peter.whorwell@smuht.nwest.nhs.uk), Olafur Palsson (opalsson@med.unc.edu), Juliette Rutten (j.m.rutten@amc.uva.nl), Emma Louise Calvert.

Figure legends

X

Figure 1. Standard Protocol Items: Recommendations for Interventional Trials (SPIRIT) Figure displaying the trial design and the outcome measurements. After screening for eligibility (t-1), children and parents sign the informed consent form and fill in the baseline questionnaire (t0). Children are then randomized in the hypnotherapy (HT) or standard medical care (SMT) group. Assessments take place before the start of treatment (baseline; t0), 6 weeks after the start of treatment (t1), 3 months after the start of treatment (t2), 6 months follow-up (t3) and 12 months follow-up (t4).

X

Figure 2. The Consolidated Standards of Reporting Trials (CONSORT) flow diagram indicating the number of participants throughout the study. After eligibility screening, children are randomized in either the hypnotherapy (HT) or standard medical care (SMT) group. Follow-up measurements take place at 6 weeks after the start of treatment (t1), 3 months after the start of treatment (t2), and 6 months follow-up (t3) and 12 months follow-up (t4).

For peer review only

21 November 2019. V2.

1. Robin, S. G., Keller, C., Zwiener, R., et al. Prevalence of pediatric functional gastrointestinal disorders utilizing the Rome IV criteria. *The Journal of pediatrics* 2018;195:134-139.

2. Kovacic, K., Williams, S., Li, et al. High prevalence of nausea in children with pain-associated functional gastrointestinal disorders: are Rome criteria applicable? *Journal of Pediatric Gastroenterology and Nutrition* 2013;*57*(3):311–5.

3. Perez, M. E., & Youssef, N. N. Dyspepsia in childhood and adolescence: insights and treatment considerations. *Current Gastroenterology Reports*, 2007;*9*(6):447–55.

4. Russell, A., Sherman, A. L., & Walker, L. S. Nausea Complicating Recurrent Abdominal Pain in Childhood Predicts Functional GI Disorders, Disability, Depression and Anxiety in Young Adulthood: Results of a Prospective Cohort Study. *Gastroenterology* 2015; 148:S–122.

5. Brook, R. A., Kleinman, N. L., Choung, R. S., et al. Functional Dyspepsia Impacts Absenteeism and Direct and Indirect Costs. *Clinical Gastroenterology and Hepatology* 2010;8: 498–503

For peer review only

References

21 November 2019. V2.

6. Hyams, J. S., Di Lorenzo, C., Saps, et al. Childhood Functional Gastrointestinal Disorders: Child/Adolescent. *Gastroenterology*, 2016;150:1456–1468.e2.

7. Madani, S., Cortes, O., & Thomas, R. Cyproheptadine Use in Children With Functional Gastrointestinal Disorders. *Journal of Pediatric Gastroenterology and Nutrition*, 2016;62: 409–413.

8. Rodriguez, L., Diaz, J., & Nurko, S. Safety and Efficacy of Cyproheptadine for Treating Dyspeptic Symptoms in Children. *The Journal of Pediatrics*, 2013;163:261–267.

9. Kellow, J. E., Azpiroz, F., Delvaux, M., et al. Applied Principles of Neurogastroenterology: Physiology/Motility Sensation. *Gastroenterology,* 2006*;*130(5):1412–1420. http://doi.org/10.1053/j.gastro.2005.08.061

10. Oudenhove, L. Van, Vandenberghe, J., Geeraerts, B., Vos, R., et al. Determinants of symptoms in functional dyspepsia: gastric sensorimotor function, psychosocial factors or somatisation? *Gut* 2008;57:1666-1673

11. Häuser, W., Hagl, M., Schmierer, A., et al. The Efficacy, Safety and Applications of Medical Hypnosis. *Deutsches Arzteblatt International*, 2016; 113: 289–96.

12. Miller, V., Carruthers, H. R., Morris, J., et al. Hypnotherapy for irritable bowel syndrome: an audit of one thousand adult patients. *Alimentary Pharmacology & Therapeutics*, 2015;*41*;844–855

13. Vlieger, A. M., Menko-Frankenhuis, C., Wolfkamp, S. C. S., et al. Hypnotherapy for children with functional abdominal pain or irritable bowel syndrome: a randomized controlled trial. *Gastroenterology*, 2007;133:1430–6

14. Richardson, J., Smith, J. E., McCall, G., Richardson, A., Hypnosis for nausea and vomiting in cancer chemotherapy: a systematic review of the research evidence. *Eur J Cancer Care (Engl)*, 2007;*16*:402–412

15. Whorwell, P. J., Houghton, L. A., Taylor, E. E., et al. Physiological effects of emotion: assessment via hypnosis. *Lancet*, 1992;340(8811):69–72.

16. Gonsalkorale, W. M., Houghton, L. A., & Whorwell, P. J. Hypnotherapy in irritable bowel syndrome: a large-scale audit of a clinical service with examination of factors influencing responsiveness. *The American Journal of Gastroenterology*, 2002;97:954–961. http://doi.org/10.1111/j.1572-0241.2002.05615.x

17. Lea, R., Houghton, L. A., Calvert, E. L., et al. Gut-focused hypnotherapy normalizes disordered rectal sensitivity in patients with irritable bowel syndrome. *Alimentary Pharmacology & Therapeutics*, 2003;*17*:635–42.

18. Lowén, M. B. O., Mayer, E. A., Sjöberg, et al. Effect of hypnotherapy and educational intervention on brain response to visceral stimulus in the irritable bowel syndrome. *Alimentary Pharmacology & Therapeutics*, 2013;*37*:1184–1197.

For peer review only

21 November 2019. V2.

19. Prior, A., Colgan, S. M., & Whorwell, P. J. Changes in rectal sensitivity after hypnotherapy in patients with irritable bowel syndrome. *Gut*, 1990;*31*:896–8

20. Rutten, J. M. T. M., Vlieger, A. M., Frankenhuis, C., et al. Home-Based Hypnotherapy Self-exercises vs Individual Hypnotherapy With a Therapist for Treatment of Pediatric Irritable Bowel Syndrome, Functional Abdominal Pain, or Functional Abdominal Pain Syndrome. *JAMA Pediatrics*, 2017:*171*;470.

21. Rutten, J. M., Vlieger, A. M., Frankenhuis, C., et al. Gut-directed hypnotherapy in children with irritable bowel syndrome or functional abdominal pain (syndrome): a randomized controlled trial on self exercises at home using CD versus individual therapy by qualified therapists. *BMC Pediatrics*, 2014;*14*(1):140

22. Vlieger, A. M., Rutten, J. M. T. M., Govers, A. M. A. P., et al. Long-term follow-up of gut-directed hypnotherapy vs. standard care in children with functional abdominal pain or irritable bowel syndrome. *The American Journal of Gastroenterology*, 2012;*107*(4):627–31.

23. Baxter, A. L., Watcha, M. F., Baxter, W. V. et al. Development and validation of a pictorial nausea rating scale for children. *Pediatrics*, 2011;*127*:e1542–9.

24. Canan, O., Ozcay, F., Ozbay-Hosnut, F., et al. Value of the Likert dyspepsia scale in differentiation of functional and organic dyspepsia in children. *Journal of Pediatric Gastroenterology and Nutrition*, 2011;*52*:392–8

25. De Luca, L., Zagari, R. M., Pozzato, P., et al. Measuring dyspepsia: a new severity index validated in Bologna. *Digestive and Liver Disease*, 2004;*36*: 806–810.

26. Ravens-Sieberer, U., Gosch, A., Rajmil, L., et al. KIDSCREEN Group. The KIDSCREEN-52 Quality of Life Measure for Children and Adolescents: Psychometric Results from a Cross-Cultural Survey in 13 European Countries. *Value in Health*, 2008;*11*:645–658.

27. Ravens-Sieberer, U., Auquier, P., Erhart, M., et al. The KIDSCREEN-27 quality of life measure for children and adolescents: psychometric results from a cross-cultural survey in 13 European countries. *Quality of Life Research*, 2007;*16*:1347-1356.

28. Muris, P., Meesters, C., & Schouten, E. A brief questionnaire of DSM-IV-defined anxiety and depression symptoms among children. *Clinical Psychology & Psychotherapy*, 2002;*9*:430–442

29. Eiser, C., & Morse, R. The measurement of quality of life in children: past and future perspectives. *Journal of Developmental and Behavioral Pediatrics : JDBP*, 2001; *22*: 248–56

30. Feeny, D., Furlong, W., Torrance, G. W., et al. Multiattribute and single-attribute utility functions for the health utilities index mark 3 system. *Medical Care*, 2002;*40*:113–28

For peer review only - http://bmjopen.bmj.com/site/about/guidelines.xhtml

21 November 2019. V2.

31. Tarride, J.-E., Burke, N., Bischof, M., et al. A review of health utilities across conditions common in paediatric and adult populations. *Health and Quality of Life Outcomes*, 2010;*8*:12.

32. Van Roijen, L., Essink-bot, M.-L., Koopmanschap, M. A., et al. Labor and Health Status in Economic Evaluation of Health Care: The Health and Labor Questionnaire. *International Journal of Technology Assessment in Health Care*, 1996;*12*:405–415.

33. Meesters, C., Muris, P., Ghys, A., et al. The Children's Somatization Inventory: further evidence for its reliability and validity in a pediatric and a community sample of Dutch children and adolescents. *Journal of Pediatric Psychology*, 2003;*28*:413–22.

34. Mangel, A., Hahn, B., Heath, A., Northcutt, A., et al. Adequate Relief as an Endpoint in Clinical Trials in Irritable Bowel Syndrome. *Journal of International Medical Research*, 1998;*26*:76–81.

35. Calvert, E. L., Houghton, L. A., Cooper, P., et al. Long-term improvement in functional dyspepsia using hypnotherapy. *Gastroenterology*, 2002;*123*:1778–1785.

36. World Medical Association. World Medical Association Declaration of Helsinki. Ethical principles for medical research involving human subjects. *Bulletin of the World Health Organization*, 2001;*79*:373.

37. Browne, P. D., Nagelkerke, S. C. J., van Etten-Jamaludin, et al. Pharmacological treatments for functional nausea and functional dyspepsia in children: a systematic review. 2018 *Expert Review of Clinical Pharmacology*, (*in press*)

38. Veldhuyzen van Zanten, S. J., Talley, N. J., Bytzer, P., et al. Design of treatment trials for functional gastrointestinal disorders. *Gut*, 1999;*45:*69–77.

39. Miele, E., Simeone, D., Marino, A., et al. Functional gastrointestinal disorders in children: an Italian prospective survey. *Pediatrics*, 2004;*114*:73-78

40. Dossett, M. L., Mu, L., Davis, R. B., et al. Patient-Provider Interactions Affect Symptoms in Gastroesophageal Reflux Disease: A Pilot Randomized, Double-Blind, Placebo-Controlled Trial. *PLOS ONE*, 2015;*10*:e0136855

613

For peer review only - http://bmjopen.bmj.com/site/about/guidelines.xhtml

	Enrolment	Allocation	During treatment		Follow-up	
**TIMEPOINT**	Before treatment (t-1)	Start of treatment (t0)	6 weeks after the start of treatment (t1)	3 months after the start of treatment (t2)	6 months follow-up (t3)	12 months follow-up (t4)
**ENROLMENT:**						
**Eligibility screen**	X					
**Informed consent**	X					
**Randomization**	X					
**INTERVENTIONS:**						
**Hypnotherapy group (HT)**		←	—	→		
**Standard medical care group (SMT)**		←	—	→		
**ASSESSMENTS:**						
Nausea and abdominal pain	X		X	X	X	X
Dyspeptic symptoms		X		X	X	X
Health related quality of life		X		X	X	X
Anxiety and Depression		X		X	X	X
Cost effectiveness/cost utility		X		X	X	X
Work absenteeism by parents and school absenteeism by children		X		X	X	X
Somatization		X		X	X	X
Adequate relief					X	X

Figure 1. Standard Protocol Items: Recommendations for Interventional Trials (SPIRIT) Figure displaying the trial design and the outcome measurements. After screening for eligibility (t-1), children and parents sign the informed consent form and fill in the baseline questionnaire (t0). Children are then randomized in the hypnotherapy (HT) or standard medical care (SMT) group. Assessments take place before the start of treatment (baseline; t0), 6 weeks after the start of treatment (t1), 3 months after the start of treatment (t2), 6 months follow-up (t3) and 12 months follow-up (t4).

215x279mm (300 x 300 DPI)

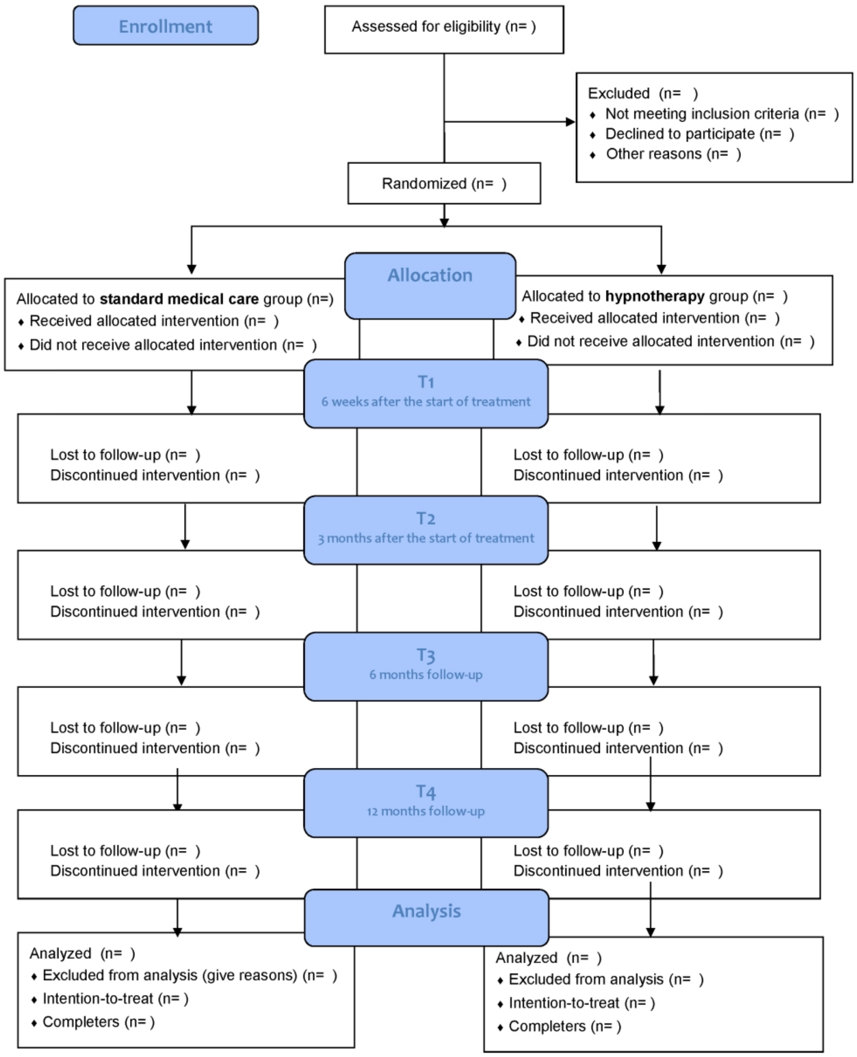

Figure 2. The Consolidated Standards of Reporting Trials (CONSORT) flow diagram indicating the number of participants throughout the study. After eligibility screening, children are randomized in either the hypnotherapy (HT) or standard medical care (SMT) group. Follow-up measurements take place at 6 weeks after the start of treatment (t1), 3 months after the start of treatment (t2), and 6 months follow-up (t3) and 12 months follow-up (t4).

215x279mm (300 x 300 DPI)

Reporting checklist for protocol of a clinical trial.

Based on the SPIRIT guidelines.

Instructions to authors

Complete this checklist by entering the page numbers from your manuscript where readers will find each of the items listed below.

Your article may not currently address all the items on the checklist. Please modify your text to include the missing information. If you are certain that an item does not apply, please write "n/a" and provide a short explanation.

Upload your completed checklist as an extra file when you submit to a journal.

In your methods section, say that you used the SPIRIT reporting guidelines, and cite them as:

Chan A-W, Tetzlaff JM, Altman DG, Laupacis A, Gøtzsche PC, Krleža-Jerić K, Hróbjartsson A, Mann H, Dickersin K, Berlin J, Doré C, Parulekar W, Summerskill W, Groves T, Schulz K, Sox H, Rockhold FW, Rennie D, Moher D. SPIRIT 2013 Statement: Defining standard protocol items for clinical trials. Ann Intern Med. 2013;158(3):200-207

		Reporting Item	Page Number
Title	#1	Descriptive title identifying the study design, population, interventions, and, if applicable, trial acronym	1
Trial registration	#2a	Trial identifier and registry name. If not yet registered, name of intended registry	2
Trial registration: data set	#2b	All items from the World Health Organization Trial Registration Data Set	3-14
Protocol version	#3	Date and version identifier	1
Funding	#4	Sources and types of financial, material, and other support	18
Roles and responsibilities: contributorship	#5a	Names, affiliations, and roles of protocol contributors	1, 18
Roles and	#5b	Name and contact information for the trial sponsor	18

For peer review only

responsibilities: sponsor contact information			
Roles and responsibilities: sponsor and funder	#5c	Role of study sponsor and funders, if any, in study design; collection, management, analysis, and interpretation of data; writing of the report; and the decision to submit the report for publication, including whether they will have ultimate authority over any of these activities	18
Roles and responsibilities: committees	#5d	Composition, roles, and responsibilities of the coordinating centre, steering committee, endpoint adjudication committee, data management team, and other individuals or groups overseeing the trial, if applicable (see Item 21a for data monitoring committee)	NA
Background and rationale	#6a	Description of research question and justification for undertaking the trial, including summary of relevant studies (published and unpublished) examining benefits and harms for each intervention	3, 4
Background and rationale: choice of comparators	#6b	Explanation for choice of comparators	5
Objectives	#7	Specific objectives or hypotheses	4
Trial design	#8	Description of trial design including type of trial (eg, parallel group, crossover, factorial, single group), allocation ratio, and framework (eg, superiority, equivalence, non-inferiority, exploratory)	5
Study setting	#9	Description of study settings (eg, community clinic, academic hospital) and list of countries where data will be collected. Reference to where list of study sites can be obtained	5
Eligibility criteria	#10	Inclusion and exclusion criteria for participants. If applicable, eligibility criteria for study centres and individuals who will perform the interventions (eg, surgeons, psychotherapists)	6
Interventions:	#11a	Interventions for each group with sufficient detail to allow	7-9

For peer review only

description		replication, including how and when they will be administered	
Interventions: modifications	#11b	Criteria for discontinuing or modifying allocated interventions for a given trial participant (eg, drug dose change in response to harms, participant request, or improving / worsening disease)	9, 15
Interventions: adherance	#11c	Strategies to improve adherence to intervention protocols, and any procedures for monitoring adherence (eg, drug tablet return; laboratory tests)	9
Interventions: concomitant care	#11d	Relevant concomitant care and interventions that are permitted or prohibited during the trial	9
Outcomes	#12	Primary, secondary, and other outcomes, including the specific measurement variable (eg, systolic blood pressure), analysis metric (eg, change from baseline, final value, time to event), method of aggregation (eg, median, proportion), and time point for each outcome. Explanation of the clinical relevance of chosen efficacy and harm outcomes is strongly recommended	9-13
Participant timeline	#13	Time schedule of enrolment, interventions (including any run-ins and washouts), assessments, and visits for participants. A schematic diagram is highly recommended (see Figure)	5
Sample size	#14	Estimated number of participants needed to achieve study objectives and how it was determined, including clinical and statistical assumptions supporting any sample size calculations	13-14
Recruitment	#15	Strategies for achieving adequate participant enrolment to reach target sample size	5
Allocation: sequence generation	#16a	Method of generating the allocation sequence (eg, computer-generated random numbers), and list of any factors for stratification. To reduce predictability of a random sequence, details of any planned restriction (eg, blocking) should be provided in a separate document that is unavailable to those who enrol participants or assign	6-7

For peer review only

		interventions	
Allocation concealment mechanism	#16b	Mechanism of implementing the allocation sequence (eg, central telephone; sequentially numbered, opaque, sealed envelopes), describing any steps to conceal the sequence until interventions are assigned	6-7
Allocation: implementation	#16c	Who will generate the allocation sequence, who will enrol participants, and who will assign participants to interventions	6-7
Blinding (masking)	#17a	Who will be blinded after assignment to interventions (eg, trial participants, care providers, outcome assessors, data analysts), and how	6-7
Blinding (masking): emergency unblinding	#17b	If blinded, circumstances under which unblinding is permissible, and procedure for revealing a participant's allocated intervention during the trial	NA
Data collection plan	#18a	Plans for assessment and collection of outcome, baseline, and other trial data, including any related processes to promote data quality (eg, duplicate measurements, training of assessors) and a description of study instruments (eg, questionnaires, laboratory tests) along with their reliability and validity, if known. Reference to where data collection forms can be found, if not in the protocol	9-13
Data collection plan: retention	#18b	Plans to promote participant retention and complete follow-up, including list of any outcome data to be collected for participants who discontinue or deviate from intervention protocols	9
Data management	#19	Plans for data entry, coding, security, and storage, including any related processes to promote data quality (eg, double data entry; range checks for data values). Reference to where details of data management procedures can be found, if not in the protocol	15
Statistics: outcomes	#20a	Statistical methods for analysing primary and secondary outcomes. Reference to where other details of the statistical analysis plan can be found, if not in the protocol	14-15

For peer review only

Statistics: additional analyses	#20b	Methods for any additional analyses (eg, subgroup and adjusted analyses)	NA
Statistics: analysis population and missing data	#20c	Definition of analysis population relating to protocol non-adherence (eg, as randomised analysis), and any statistical methods to handle missing data (eg, multiple imputation)	14
Data monitoring: formal committee	#21a	Composition of data monitoring committee (DMC); summary of its role and reporting structure; statement of whether it is independent from the sponsor and competing interests; and reference to where further details about its charter can be found, if not in the protocol. Alternatively, an explanation of why a DMC is not needed	15
Data monitoring: interim analysis	#21b	Description of any interim analyses and stopping guidelines, including who will have access to these interim results and make the final decision to terminate the trial	NA
Harms	#22	Plans for collecting, assessing, reporting, and managing solicited and spontaneously reported adverse events and other unintended effects of trial interventions or trial conduct	15
Auditing	#23	Frequency and procedures for auditing trial conduct, if any, and whether the process will be independent from investigators and the sponsor	15
Research ethics approval	#24	Plans for seeking research ethics committee / institutional review board (REC / IRB) approval	P1
Protocol amendments	#25	Plans for communicating important protocol modifications (eg, changes to eligibility criteria, outcomes, analyses) to relevant parties (eg, investigators, REC / IRBs, trial participants, trial registries, journals, regulators)	14
Consent or assent	#26a	Who will obtain informed consent or assent from potential trial participants or authorised surrogates, and how (see Item 32)	19
Consent or assent: ancillary studies	#26b	Additional consent provisions for collection and use of participant data and biological specimens in ancillary studies, if applicable	NA

For peer review only

Confidentiality	#27	How personal information about potential and enrolled participants will be collected, shared, and maintained in order to protect confidentiality before, during, and after the trial	15
Declaration of interests	#28	Financial and other competing interests for principal investigators for the overall trial and each study site	18
Data access	#29	Statement of who will have access to the final trial dataset, and disclosure of contractual agreements that limit such access for investigators	15
Ancillary and post trial care	#30	Provisions, if any, for ancillary and post-trial care, and for compensation to those who suffer harm from trial participation	15
Dissemination policy: trial results	#31a	Plans for investigators and sponsor to communicate trial results to participants, healthcare professionals, the public, and other relevant groups (eg, via publication, reporting in results databases, or other data sharing arrangements), including any publication restrictions	16
Dissemination policy: authorship	#31b	Authorship eligibility guidelines and any intended use of professional writers	NA
Dissemination policy: reproducible research	#31c	Plans, if any, for granting public access to the full protocol, participant-level dataset, and statistical code	NA
Informed consent materials	#32	Model consent form and other related documentation given to participants and authorised surrogates	NA
Biological specimens	#33	Plans for collection, laboratory evaluation, and storage of biological specimens for genetic or molecular analysis in the current trial and for future use in ancillary studies, if applicable	NA

The SPIRIT checklist is distributed under the terms of the Creative Commons Attribution License CC-BY-ND 3.0. This checklist was completed on 19. June 2018 using http://www.goodreports.org/, a tool made by the EQUATOR Network in collaboration with Penelope.ai

For peer review only
